# 3D-printed porous functional composite scaffolds with polydopamine decoration for bone regeneration

**DOI:** 10.1093/rb/rbad062

**Published:** 2023-06-21

**Authors:** Jin Qi, Yili Wang, Liping Chen, Linjie Chen, Feng Wen, Lijiang Huang, Pfukwa Rueben, Chunwu Zhang, Huaqiong Li

**Affiliations:** Department of Orthopaedics, Joint Centre of Translational Medicine, The First Affiliated Hospital of Wenzhou Medical University, Wenzhou, Zhejiang 325035, P. R. China; Joint Centre of Translational Medicine, Zhejiang Engineering Research Center for Tissue Repair Materials, Wenzhou Institute, University of Chinese Academy of Sciences, Wenzhou, Zhejiang 325011, P. R. China; University of Chinese Academy of Sciences, Beijing 100049, P. R. China; Department of Orthopaedics, Joint Centre of Translational Medicine, The First Affiliated Hospital of Wenzhou Medical University, Wenzhou, Zhejiang 325035, P. R. China; Joint Centre of Translational Medicine, Zhejiang Engineering Research Center for Tissue Repair Materials, Wenzhou Institute, University of Chinese Academy of Sciences, Wenzhou, Zhejiang 325011, P. R. China; Department of Orthopaedics, Joint Centre of Translational Medicine, The First Affiliated Hospital of Wenzhou Medical University, Wenzhou, Zhejiang 325035, P. R. China; Joint Centre of Translational Medicine, Zhejiang Engineering Research Center for Tissue Repair Materials, Wenzhou Institute, University of Chinese Academy of Sciences, Wenzhou, Zhejiang 325011, P. R. China; The Second Affiliated Hospital of Wenzhou Medical University, Wenzhou, Zhejiang 325035, P. R. China; Department of Orthopaedics, Joint Centre of Translational Medicine, The First Affiliated Hospital of Wenzhou Medical University, Wenzhou, Zhejiang 325035, P. R. China; Joint Centre of Translational Medicine, Zhejiang Engineering Research Center for Tissue Repair Materials, Wenzhou Institute, University of Chinese Academy of Sciences, Wenzhou, Zhejiang 325011, P. R. China; The Affiliated Xiangshan Hospital of Wenzhou Medical University, Ningbo, Zhejiang 315700, P. R. China; Department of Chemistry and Polymer Science, Stellenbosch University, Matieland, Stellenbosch 7602, South Africa; Department of Orthopaedics, Joint Centre of Translational Medicine, The First Affiliated Hospital of Wenzhou Medical University, Wenzhou, Zhejiang 325035, P. R. China; Department of Orthopaedics, Joint Centre of Translational Medicine, The First Affiliated Hospital of Wenzhou Medical University, Wenzhou, Zhejiang 325035, P. R. China; Joint Centre of Translational Medicine, Zhejiang Engineering Research Center for Tissue Repair Materials, Wenzhou Institute, University of Chinese Academy of Sciences, Wenzhou, Zhejiang 325011, P. R. China

**Keywords:** 3D printing, bioactive glass composites, polydopamine, angiogenesis, bone regeneration

## Abstract

Large size bone defects affect human health and remain a worldwide health problem that needs to be solved immediately. 3D printing technology has attracted substantial attention for preparing penetrable multifunctional scaffolds to promote bone reconditioning and regeneration. Inspired by the spongy structure of natural bone, novel porous degradable scaffolds have been printed using polymerization of lactide and caprolactone (PLCL) and bioactive glass 45S5 (BG), and polydopamine (PDA) was used to decorate the PLCL/BG scaffolds. The physicochemical properties of the PLCL/BG and PLCL/BG/PDA scaffolds were measured, and their osteogenic and angiogenic effects were characterized through a series of experiments both *in vitro* and *in vivo*. The results show that the PLCL/BG2/PDA scaffold possessed a good compression modulus and brilliant hydrophilicity. The proliferation, adhesion and osteogenesis of hBMSCs were improved in the PDA coating groups, which exhibited the best performance. The results of the SD rat cranium defect model indicate that PLCL/BG2/PDA obviously promoted osteointegration, which was further confirmed through immunohistochemical staining. Therefore, PDA decoration and the sustained release of bioactive ions (Ca, Si, P) from BG in the 3D-printed PLCL/BG2/PDA scaffold could improve surface bioactivity and promote better osteogenesis and angiogenesis, which may provide a valuable basis for customized implants in extensive bone defect repair applications.

## Graphical abstract

**Figure:**
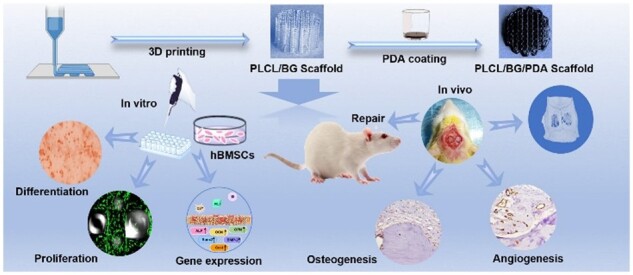

## Introduction

Bone plays an important role in providing mechanical support and hematopoiesis as well as protecting the organs inside the body as a vital tissue/organ of the human body. Reconstruction of critical size bone defects resulting from trauma, accidents and bone necrosis has historically been a great challenge for patients and surgeons worldwide [1]. Although autologous bone grafts and allografts have shown favorable potential to repair many bone defects in clinical treatment, size mismatch, immune rejection, a shortage of bone donors and infected bone limit their further applications [2]. In recent years, with the development of 3D printing technology, 3D-printed porous structures have attracted much attention in the tissue engineering field due to their high precision and personalized customization [3], and has provided a solution to the above problems. It is well known that native bone is mainly composed of compact bone and spongy bone, and bone essentially involves cells and a plentiful extracellular matrix [4]. This highly ordered hierarchical structure enhances the adaptability of the scaffolds [5]. Therefore, developing biomimetic 3D-printed porous structures with complex gradient architectures analogous to natural bone is necessary [6]. With the progress of tissue engineering, various 3D printing technologies, such as selective laser sintering [7], stereolithography [8] and fused deposition modeling [9], have been used to prepare porous scaffolds for bone defect repair. In recent years, 3D bioprinting technology has attracted great attention for establishing bionic systems. It precisely integrates biomaterials and bioactive factors as well as multiple tissue cell additions [10, 11]. Based on this versatility, 3D bioprinting has been used to construct artificial lung, muscle and heart complex tissue *in vitro* [12, 13]. Therefore, 3D printing can be used to precisely mimic the porous structure of bone by designing the consistent assignment of tissue cells, transport of nutrients and excretion of metabolic wastes [14].

Bone tissue engineering scaffolds with controllable degradation rates, tunable biological performances and adjustable mechanical strength can be prepared by hydrogel composites, bioceramic powders, polymer melts and polymer/bioceramic composites [10, 15, 16, 17, 18, 19]. Among different bioactive materials, calcium phosphate bioceramics such as bioactive glass, hydroxyapatite (HA) and tricalcium phosphate are the most commonly used inorganic materials in making osteoconductive bone tissue engineering scaffolds [4, 20, 21], and polymers such as polylactic acid, polycaprolactone (PCL), poly(lactide-co-glycolide) and polytrimethylene carbonate are the most frequently used 3D printing materials for bone tissue engineering [22–25]. Because aliphatic polyester and bioceramics have multitudinous disadvantages, including acidic degraded products, poor bone-bonding bioactivity and osteogenic activity of the pure polymers, single bioceramics are brittle and cannot bear weight simultaneously [26]. Scaffolds made of polymers or inorganic particles alone lack sufficient bone-forming ability. Compared with monophasic scaffolds for repairing defect bone tissue, 3D-printed organ/inorganic composite structures are more likely to resolve the natural deficiencies of bioceramic powders and polymers [27]. In addition, the 3D printing technique has benefits related to the precise procedure, including accurate size and different controllable shapes of scaffolds [25]. Therefore, employing a 3D printing model to build osteoinductive porous scaffolds is expected to prevent the inadequate mechanical combination and compound fabrication process of the multiphasic composite scaffolds reported previously. In addition, it was reported that 3D-printed scaffolds can provide lively exogenous cells for the necrosis area, where the cell concentration, cell metabolism and migration ability can be adjusted by the porous implant [5]. Therefore, the configuration of organic/inorganic spongey scaffolds that can induce angiogenic osteogenesis may be dedicated to repairing bone defects. Polymer/bioceramics are competent in regulating stem cell differentiation proliferation and facilitating defective tissue regeneration. It has been reported that porous PCL/SrCuSi_4_O_10_ scaffolds can increase osteogenic activity [4]. Ca and P bioceramics have been reported to release multiple ions (Ca, P, Si) with different bioactivities to initiate tissue repair and regeneration [4, 28, 29]. Studies have proven that these ions show a synergistic effect with Si ions to accelerate bone tissue regeneration [10, 30, 31].

Polymerization of lactide and caprolactone (PLCL) can result in a copolymer prepared from lactide and caprolactone monomers. The degradation rate of PLCL could be controlled by the monomer proportion to reduce the acid degradation products. Because of its marvelous biodegradability and biocompatibility, it has been studied for cell adhesion and tissue defect repair [32]. Because of its excellent performance in inducing cell adhesion, good biocompatibility and hydrophilicity, auto-polymerized polydopamine (PDA) has been proverbially used in the surface decoration of biomaterials [33, 34]. Many vivacious groups, such as quinone and *o*-phenol hydroxyl groups, are present on the PDA surface. PDA can be closely bound with nearly all types of external components to enhance the stability of composites, which is attributed to its unique chemical structures [35–37]. Recent studies have found that PDA can both facilitate the formation of calcium phosphate mineralization and significantly stimulate the adhesion and differentiation of bone-related cells on the surface of tissue-engineered scaffolds [33, 38, 39]. According to the concept of ‘background adhesion’, it reported that cells adhered to the PDA coating better than untreated surface, which influenced mobility and eventually proliferation. Hence, the cell behaviors on the PDA coating are reasonable and complicated [38].

To sum up, there were various organic–inorganic hybrid scaffolds for different tissue regeneration have been developed by many research groups [6, 15, 16, 17, 18, 19, 32, 33, 34, 35]. The combination of PLCL/BG scaffold and PDA may be an effective method to facilitate bone defect repair in theory. However, the biocompatibility of PLCL/BG/PDA composites have yet to be fully studied. Additionally, whether the PDA coating is suitable for PLCL/BG composites porous structure in bone regeneration is still unknown. In the present work, tailorable bone tissue engineering porous composite scaffolds with outstanding mechanical properties, adjustable degradation rates, connected porous structures and favorable osteoinductivity and osteoconductivity were produced via 3D printing technology of bioactive glass 45S5 (BG) and PLCL blends. The coating of PDA on the surface further enhanced the hydrophilicity, cell adhesion and proliferation of the porous scaffolds. The physical and chemical properties of the biomaterials were characterized. The osteogenesis and angiogenesis capabilities of the PLCL/BG/PDA composite scaffold were studied both *in vitro* and *in vivo*. The results demonstrate that the PLCL scaffolds with bioactive glass and PDA could greatly facilitate bone regeneration.

## Materials and methods

### Materials

PLCL was purchased from Daigang Biomaterials (Jinan, China). The ratio of the copolymer composition (LA:CL) was 80:20 (mol:mol) with a Mw of 215.56 kDa. Implant grade 45S5 (BG) was purchased from Dongguan Hannuo Biotechnology Co., Ltd. Other agents were all of analytical grade.

### Fabrication of 3D PLCL/BG scaffolds and PDA treatment

Before 3D printing, we first prepared PLCL/BG composites. Briefly, PLCL and BG with varying weight ratios (100:0, 100:2, 100:5, 100:8) were prepared. PLCL was dissolved in DCM at room temperature, and then the BG particles were added to the PLCL solution under ultrasonic dispersion (10% w/v). Finally, PLCL/BG composites were obtained with absolute ethanol precipitation and dried in a vacuum oven at 40°C for 48 h. Subsequently, the composite scaffold model was designed by using CAD/CAM software and exported in STL format. A bioplotter printer was used to prepare the desired scaffolds. A metal nozzle (22G) was employed. The target scaffolds were named PLCL, PLCL/BG2, PLCL/BG5 and PLCL/BG8 according to the BG content in the composite scaffold.

To prepare the PDA-coated PLCL scaffold and PLCL/BG composite scaffolds, an immersion procedure was used [9]. Dopamine hydrochloride was added to Tris–HCl (pH = 8.5) to obtain the PDA solution. Both the PLCL scaffold and PLCL/BG composite scaffolds were added to PDA solution and placed on a shaker for reaction for 24 h. Finally, after ultrasonic cleaning, the obtained PDA coating scaffolds were dried for the following experiment.

### Material characterization

The chemical structure of the PLCL composites was tested by FTIR technology (Tensor ll). After uniform sputtering with a gold layer on the scaffold, SEM (SU8010, Japan) with an acceleration voltage of 3 kV was used to observe the morphologies of the porous composite scaffolds. An electromechanical universal testing machine (5944, Instron, USA 23 ± 2°C) was applied to investigate the compressive strength of various porous structures. The porosity of the porous scaffolds was calculated according to reference [40]. TGA (4000, PerkinElmer, USA) was used to determine the stability of the materials. A heating rate of 10°C/min was used to increase the temperature from 50°C to 800°C under a nitrogen atmosphere. The change in the surface element state of the samples was observed by using XPS spectroscopy (Thermo Fisher, Escalab 250Xi) with monochromatic Al Kα X-rays. X-ray diffraction (XRD, Philips, X’ Pert Pro, Cu Kα) with a scan range from 5° to 85° and voltage of 40 kV was used to study the crystallization behavior of the PLCL/BG composites. DSC (DSC 8000, PerkinElmer) with a nitrogen atmosphere rate of 50 ml/min was used to test the thermal properties of the samples. The pH value was also measured by a pH meter (OHAUS Starter2100). The water contact angle was used to evaluate the hydrophilicity of the porous structure at room temperature. Loading of distilled water using a Theta contact angle system (Bioolin, Sweden) was performed with 50% humidity.

### Degradation evaluation in vitro

PLCL/BG composite scaffolds were immersed in inert plastic test tubes containing 5 ml of PBS, which was buffered to physiological pH 7.40 and were placed on a shaker at 37°C for four time periods, including 2, 4, 8 and 12 weeks.

The degradation properties of the pure PLCL and PLCL/BG porous composite scaffolds were tested after immersion. The ratio of water absorption and weight loss was calculated according to reference [41]. The pH value of the degradation medium, compressive strength and surface morphology of the scaffolds were measured.

### In vitro cell experiments

#### Cell culture

hBMSCs were purchased from Oricell^®^ (Cyagen, CA, China). Mesenchymal stem cell medium with 10% fetal bovine serum and a 1% penicillin mixture was used to culture the cells. Every 2–3 days, the culture medium was changed. hBMSCs were grown until they reached 80–90% confluence. Trypsin–EDTA was used to digest the cells in the tissue flask bottles, which were then placed in another Petri dish. Cells from Passages 2 to 5 were used for further experimentation. All the cells were placed in a constant temperature incubator with 5% CO_2_ at 37°C for subculture.

#### Cytotoxicity assay

Before the cell seeding procedure, scaffolds with a diameter of 8 mm and height of 2 mm were sterilized with ethylene oxide gas and then placed in a 48-well cell culture plate. For cell toxicity assessment, hBMCSs were cultured on 3D-printed porous scaffolds and PDA treatment scaffolds for 1, 3 and 7 days. The cell density of seeding was 2.5 × 10^5^ cells/well. Cytotoxicity was assessed using the CCK-8 assay kit and a multifunction microplate reader (BioTek, EPOCH/2) at an absorbance of 450 nm.

#### Cell adhesion assay

The hBMSCs and scaffolds were cultured for 3, 7 and 14 days in stem cell culture medium. At different culture time points, the scaffolds were removed and washed three times with sterile PBS. A 2.5% glutaraldehyde solution was used to fix the scaffolds in cacodylate buffer for 1 h, and the scaffolds were dehydrated with graded ethanol for 15 min each. Finally, the scaffolds were dried, and the interaction between the cells and scaffolds was observed by a SU8010 scanning electron microscope (HITACHI, Japan).

#### Confocal microscopy analyses (live/dead assay)

Cell viability and proliferation with the porous scaffolds after coculture for 1, 3, 7 and 14 days were evaluated by a live/dead assay. At different time points, the scaffolds were removed, washed with sterile PBS, and stained with ethidium homodimer-1 (EthD-1, 2 µl/ml) and Calcein AM (0.5 µl/ml) at 37°C for 15 min. Then, the scaffolds were cleaned with sterile PBS two times and tested by a laser scanning confocal microscope (AI, Nikon, Japan).

#### Cellular alkaline phosphatase activity assay, PicoGreen assay and alizarin red staining assay

hBMCSs were cultured with porous scaffolds as mentioned above. Various scaffolds were cocultured with cells for 7, 14 and 21 days. Lysate was used to lyse the cells at different time points. The DNA amount and alkaline phosphatase (ALP) activity were measured using a PicoGreen assay and ALP activity kit (Beyotime Biotechnology), respectively. The mineralized nodules in each scaffold were measured by an alizarin red (AR) staining kit (Beyotime Biotech Inc., China).

#### Quantitative real-time PCR assay

The osteogenic differentiation of hBMSCs was further studied. The expression of osteogenic-related genes, including Runx2, Col-1, OCN, CPN, BMP2 and ALP, was measured by real-time polymerase chain reaction (RT-PCR), and the coculture time of the scaffold and cells was 7, 14 and 21 days, respectively. A 20 µl system was used for the subsequent experiment. The reagent used to isolate the total RNA was purchased from Beyotime. The concentration of the isolated RNA was measured using a spectrophotometer (LightCycler^®^ 480 II). The primers of the osteogenic-related genes are listed in Table 1.

**Table 1.** Primer sequences

Gene sequence		Primer sequence (5′–3′)
GAPDH	F	CTTTGGTATCGTGGAAGGACTC
	R	GTAGAGGCAGGGATGATGTTCT
ALP	F	GTATCGGCAGCAGTCAGCAGTG
	R	TCCAGGCAGGCGGCGAAG
OCN	F	GTGACGAGTTGGCTGACC
	R	TGGAGAGGAGCAGAACTGG
OPN	F	CATACAAGGCCATCCCCGTT
	R	TGGGTTTCAGCACTCTGGTC
COL-I	F	TGGAGCAAGAGGCGAGAG
	R	CACCAGCATCACCCTTAGC
RUNX2	F	CCCTGAACTCTGCACCAAGT
	R	GGCTCAGGTAGGAGGGGTAA
BMP2	F	TCCATGTGGACGCTCTTTCA
	R	AGCAGCAACGCTAGAAGACA

### In vivo bone regeneration

#### Ethics statement

The SD rats (≈320–360 g) were provided by Zhejiang Provincial Laboratory Animal Center. According to the Laboratory Animal Care and Use Guidelines, all SD rats were treated ethically. All SD rat surgical processes were carried out according to agreements authorized by the Animal Care and Use Committee of Wenzhou Institute University of Chinese Academy of Science, Zhejiang, China (Approval number: WIUCAS22021104).

#### SD rat skull defect model

The scaffolds were sterilized using ethylene oxide gas. The skull defects were created as described in reference [42]. After anesthesia with pentobarbital, iodophor disinfection, various round craniotomy defects (diameter: 6.0 mm) were established by using a stainless-steel bone drill. The cranial defects of the animals were rinsed with normal saline and filled with sterilized scaffolds (Φ 6.0 mm × 0.672 mm). Finally, surgical suture was used to suture the wound.

Micro-CT analysis (SkyScan 1176, Bruker, Germany) was conducted after the skull tissue was collected after 4, 8 and 12 weeks, and corresponding histomorphometric analyses were conducted. The scanning was processed at a resolution of 18 µm, and 3D Creator software was used to reconstruct tomograms to obtain the images of the skull. Bone-related parameters, such as the trabecular separation (Tb.Sp), trabecular thickness (Tb.Th), bone volume/tissue volume (BV/TV) and bone mineral density (BMD), were measured by CTAn image analysis software according to the micro-CT images obtained above.

For further histological analysis, the collected skull tissue at different cycles (4, 8 and 12 weeks) was fixed with paraformaldehyde and decalcified with 10% EDTA. Paraffin was applied to embed the bone tissue. The embedded tissue was sectioned at a 5-μm thickness in the sagittal direction by a slicer. Both the control group and experimental group were stained with hematoxylin and eosin (H&E) and Masson’s trichrome (MT) to evaluate new bone regeneration in the defect area. Furthermore, immunohistochemical (IHC) staining of angiogenesis-related CD31 and osteogenesis-related osteocalcin OCN was used to measure angiogenesis and osteogenesis in the skull defects, respectively. IHC was semi-quantified by calculating the fraction of positively stained areas in ROI Image J (1.80-172, NIH, UAS).

### Statistical analysis

All data from this study are shown as the means with standard deviations (*n* ≥ 3). One-way ANOVA tests via Student’s *t* tests were conducted, and *P *<* *0.05 was considered a significant difference. (*P < 0.05, **P < 0.01 and ***P < 0.001).

## Results and discussion

### 3D-printed PLCL/BG composite scaffolds

**Figure 1.**
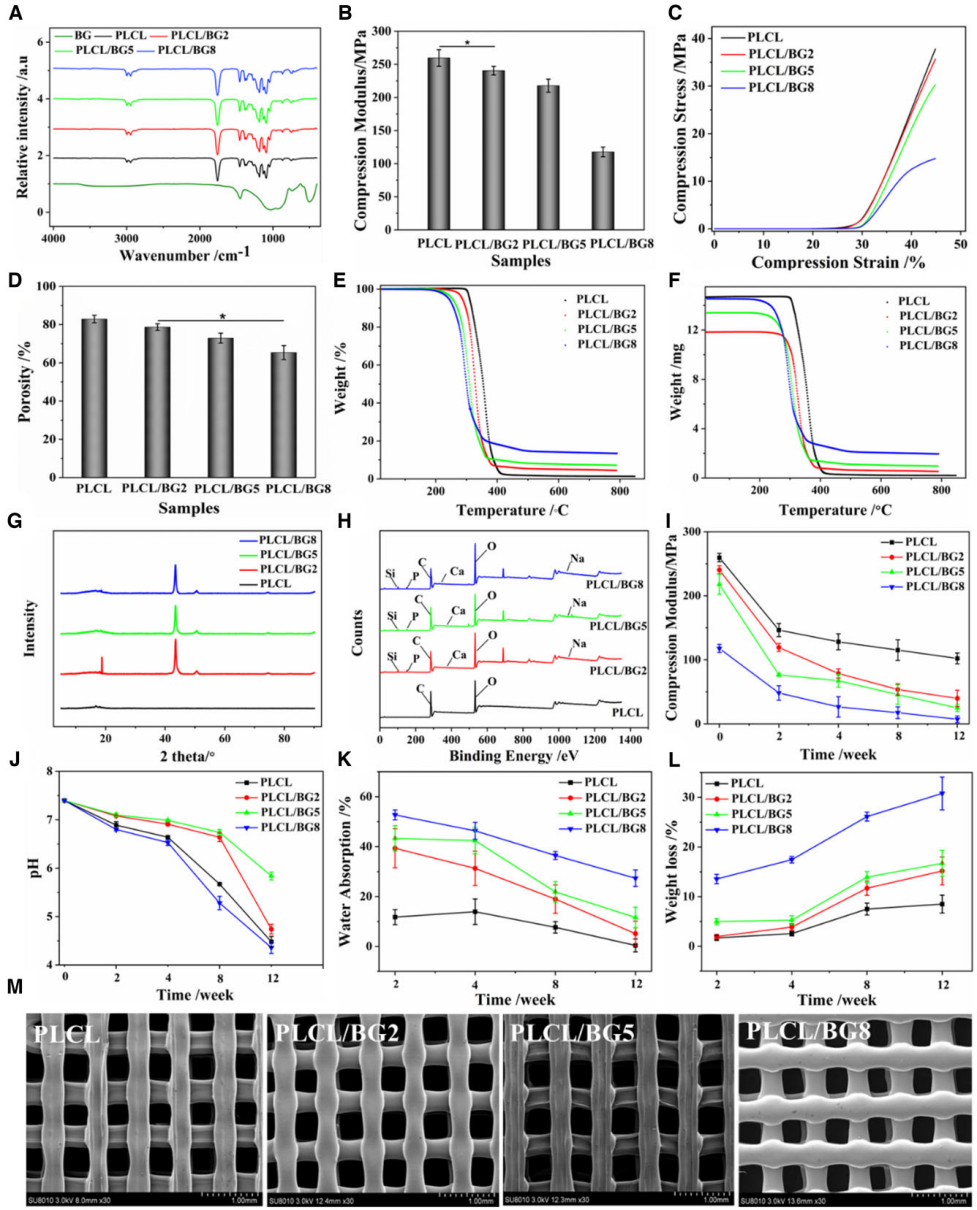
Characterization of 3D-printed PLCL/BG composite scaffolds. (**A**) FTIR spectra of BG, PLCL and PLCL/BG composites. (**B**) Compressive modulus. (**C**) Compressive stress. (**D**) Porosity. (**E**, **F**) TGA test. (**G**) XRD spectrum. (**H**) X-ray photoelectron spectroscopy. Degradation properties of PLCL/BG composite scaffolds after degradation for various cycles *in vitro*. (**I**) Compressive modulus. (**J**) pH value of the PLCL/BG scaffold degradation liquid. (**K**) Water absorption. (**L**) Weight loss. (**M**) SEM images of PLCL/BG composite scaffolds (*P < 0.05).

Figure 1a shows the infrared spectra of pure BG, PLCL and four PLCL/BG composites. BG showed strong transmittance peaks at 3513.8, 916.28–1057.56, 509.51 cm^−1^. The peak at 3513.8 cm^−1^ was attributed to the O–H and the other two belong to Si–O–Si. And the peak at 729.31 cm^−1^ was attributed to the Si–O. The peaks at 598 and 916.28–1057.56 cm^−1^ were attributed to the P–O, which overlapped with Si–O–Si in part. Due to both of PLCL and PLCL/BG composites had similar functional groups, peaks at 1756.9 and 1364.41–1457.18 cm^−1^ were attributed C=O and C–O, respectively. The peaks at 2935.78–2998.57 cm^−1^ could be assigned to C–C and the 689.44 cm^−1^ was Si–C stretching vibration. However, the absorption peaks of the PLCL/BG composites and PLCL did not shift significantly in the spectrum, and no new characteristic absorption peaks appeared. This was observed because the PLCL proportion in the composite was high, and the blends were only physically bound. The mechanical properties of porous scaffolds are important parameters for bone defect repair materials, as shown in Fig. 1b and c. With increasing BG content, the mechanical properties of the binary composite scaffolds decreased. When the addition of BG was low, it could be uniformly dispersed in the organic matrix, which hindered the movement of molecular segments of PLCL and demonstrated improvement of the compression performance on the macroscopic layer. However, since the size of BG was ∼5 µm, the reinforcing effect could not be achieved, and the compressive modulus of the PLCL/BG composite scaffolds declined. When the content of BG exceeded 8%, a large number of inorganic particles agglomerated and could not be uniformly dispersed in the organic phase, which reduced the physical bonding points of PLCL and BG, resulting in poor combination of the two phases of the scaffold and uneven stress loading, which significantly reduced its mechanical strength. When the added amount of BG in the system was 2%, the compressive modulus of the scaffold was almost the same as that of the PLCL scaffold, which was an ideal scaffold structure. Therefore, it was determined that the optimal addition of inorganic particles was 2%. It is well known that porosity has a certain influence on the mechanical properties of scaffolds. In addition, when the scaffold is implanted in the body, the higher porosity is conducive to the transport of nutrients and the discharge of metabolites, and is more conducive to vascularization and new tissue growth [14, 43]. Figure 1d presents the porosity of the PLCL/BG composite scaffolds. The porosity of the porous structure tended to decrease as the proportion of BG increases. When the composites were heated and melted, the composites with a high content of BG had better fluidity, the thickness of the pore wall became larger after extrusion, and the pore diameter decreased slightly. This eventually resulted in a decrease in the porosity of the scaffold. The DSC parameters of the PLCL and PLCL/BG composites are listed in Supplementary Table S1. The glass transition temperature means that the polymer segments start to move, the value of Tg increases, and the resistance of the segment movement is obvious. When the BG content increased, the Tg of the composite decreased and the Tm declined, indicating that BG can promote the molecular chain. The movement of the segment improves the compatibility between inorganic particles and polymers. Figure 1e and f shows the weight loss of PLCL, PLCL/BG2, PLCL/BG5 and PLCL/BG10, which were 99.079% and 95.781%, 93.037% and 86.560%, respectively. In addition, when BG was added, the decomposition temperature of the composites decreased, indicating that the thermal stability of the composites decreased when the BG content was higher (Supplementary Table S2). The XRD test showed that PLCL without inorganic particles showed a gentle bread-like peak shape (Fig. 1g), but its diffraction peak was similar to that of semicircle, and its characteristic crystal peak was at 2 theta 16.41° and 18.69°. Because of its low crystallinity, it was not obvious. After adding BG, the diffraction peak of the composites was significantly enhanced, and the diffraction peaks at 26.33°, 43.39° and 50.61° were consistent with inorganic particles, indicating that the crystallinity of the PLCL/BG composites was stronger than that of PLCL, demonstrating that BG could promote the crystallization of PLCL. The XPS spectra of the PLCL and PLCL/BG composites are presented in Fig. 1h. There were only two elements, C and O, on the pure PLCL curve. The PLCL/BG composites had six elements, including C, O, P, Ca, Si and Na. Due to the small addition ratio of BG, some element peaks are not obvious in the spectrum. The magnified images of all elements showed in Supplementary Fig. S2c. Supplementary Figure S3 shows that although the amount of BG added was small, it was evenly distributed in the scaffold. And elements contained in the composite scaffolds have been detected. These results indicate that the inorganic particles are effectively blended with PLCL.

Figure 1i shows that the mechanical strength of all porous scaffolds decreased with the degradation time. However, the compressive modulus of the PLCL scaffolds attenuated slowly with prolonged time. The dissolution of inorganic particles and the interfacial breakdown between the two phases might result in defects in the composites. The compressive modulus of the porous scaffolds of the PLCL/BG scaffolds decreased faster than that of the PLCL group with prolonged time. Especially when the content of inorganic particles was high, the compactness of the composite porous scaffold was greatly reduced, and the loss of compressive strength was increased. The degradation of the implantable material will change the pH value around it, which also has some effects on the surrounding tissue, so it is necessary to test the pH value of the degradation of porous scaffolds soaked in PBS. The pH value of the degradation solution decreased with prolonged time (Fig. 1j). This occurs because during the soaking process, the PLCL ester bond is broken and, as the degradation progressed, the carboxyl end increased, resulting in an autocatalysis phenomenon [44]. The solution pH values of the PLCL/BG2 and PLCL/BG5 scaffolds were higher than those of the PLCL scaffolds because the apatite formed by the dissolution of weakly alkaline inorganic particles and surface mineralization neutralized the acidic degradation products of PLCL. At the early stage of degradation, the pH value change of the composite scaffold degradation solution was not noticeable. However, the scaffolds had defects in the degradation process, and a large number of inorganic particles were dissolved, which accelerated the degradation of the scaffolds, and could not neutralize the acidic degradation products of PLCL, causing the pH values of the composite scaffold degradation solution to decline significantly. However, low pH may attenuate biomineralization process and further bone formation. It is reported that adjusting the pH value of the degradation process of implant materials by increasing the amounts of inorganic particles added to the composite or the composition of polymer copolymers [40, 45, 46]. For bone repair implants, physiological stability after implantation is crucial for the bone repair process, and the degradation rate of the scaffold matches the bone growth rate, improving subsequent bone remodeling to achieve functional recovery [44]. The water absorption rate of the PLCL/BG composite scaffolds at different times was higher than that of the PLCL scaffolds (Fig. 1k), which indicates that PLCL degraded slowly. The weight loss of all porous scaffolds increased continuously with degradation, and the weight loss rate of the PLCL scaffolds was lower than that of the porous structures (Fig. 1l), which also indicates that PLCL degraded slowly. The mass loss of the composite scaffolds in the later stage was slow, mainly due to the continuous mineralization of bone-like HA on the surface of scaffolds. Apatite deposition occurred on the surface of the porous structure, which increased the weight of the scaffold. Furthermore, the generated apatite deposits could alleviate the continuous degradation of the scaffold. When the content of BG was <5%, the BG and PLCL firmly combined, and the apatite deposition layer formed and retarded the degradation rate of the scaffold. In contrast, when the content of BG was more than 5%, the inorganic particles were easily peeled off, and a slight effective apatite layer formed, resulting in an increase in the weight loss of the scaffolds. As degradation proceeded, defects such as small holes and cracks appeared on the surface of the scaffold (Supplementary Fig. S1). The number and size of pores on the surface of the scaffold increased gradually during degradation. This was especially true for the PLCL/BG8 group, which showed larger defects, indicating that a high content of BG accelerated the degradation of PLCL at the late stage of degradation. From the above results, it could be judged that the poor degradability of pure PLCL might limit new bone ingrowth into the porous structure after implantation into the bone defect area; however, the addition of BG could control the degradation rate of PLCL, which might be appropriate to satisfy the degradability requirement of bone repair materials in the clinic in the future [38]. Before soaking in PBS, the surface of all scaffolds was smooth and flat without cracks (Fig. 1m). PLCL/BG composite scaffolds with different BG contents prepared by the 3D printing method have a good connected porous structure and relatively uniform pore size. With increasing proportion of inorganic particles in the system, the pore diameter gradually decreased, and the thickness of the pore wall gradually increased. When the content of BG was low, the inorganic particles were uniformly dispersed in the scaffold, and the pore size of the porous scaffold was larger. When the addition of BG exceeded 8%, the composite pore wall was thicker, the pore diameter decreased, and the surface was rough. When the amount of BG added to the system was 2 wt%, the surface of the composite scaffold was smooth, the two phases combined well, the inorganic particles were uniformly dispersed, and the thickness of the pore wall and the pore diameter were uniform, creating an ideal scaffold structure. This was consistent with the results of the mechanical strength analysis.

### PDA decoration of PLCL/BG composite scaffolds

**Figure 2.**
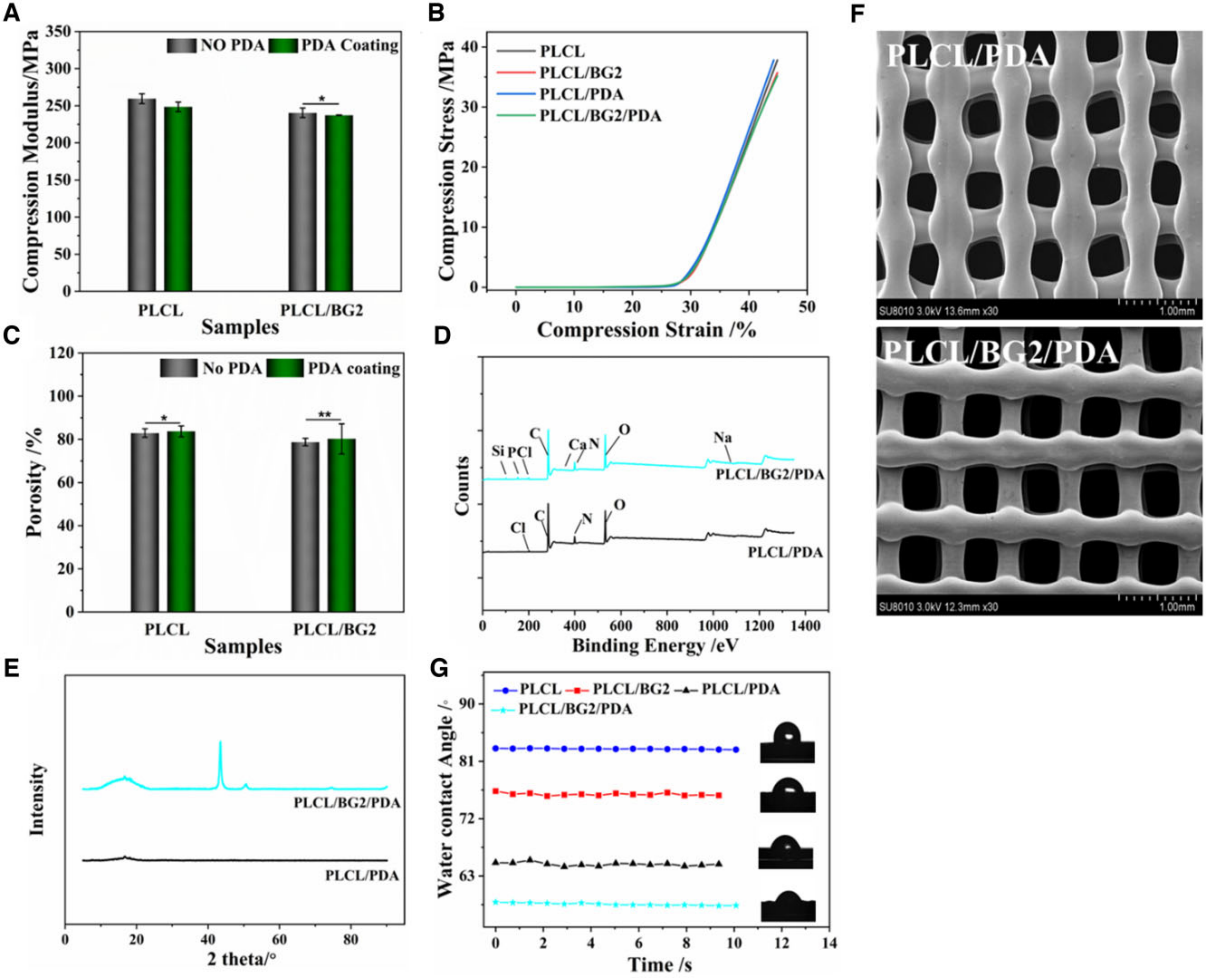
Physical and chemical properties of 3D-printed PLCL/BG composite scaffolds after PDA decoration. (**A**, **B**) Compressive modulus and compressive stress, respectively. (**C**) Porosity. (**D**) XRD. (**E**) X-ray photoelectron spectroscopy. (**F**) SEM of micrographs. (**G**) WAC measurement. (*P < 0.05, **P < 0.01).

The compressive properties of the PLCL and PLCL/BG2 scaffolds before and after PDA coating were not obviously different (Fig. 2a and b). It suggests that PDA treatment did not affect the mechanical properties of the porous scaffolds. The porosity of the porous scaffold was also not distinctly varied after PDA decoration (Fig. 2c). The XPS spectra of the PDA-processed scaffolds are presented in Fig. 2d and Supplementary Fig. S2d. After PDA treatment, the peaks of C elements in all materials increased, and two elements, N and Cl, appeared (Supplementary Fig. S2d), which reveals that PDA was successfully grafted to the surfaces of the PLCL and PLCL/BG2 composite scaffolds. The XRD test showed that PLCL/PDA without inorganic particles showed a gentle bread-like peak shape (Fig. 2e), and its characteristic crystal peak was at 2 theta 16.83° and 18.20°. For the PLCL/BG composites, the diffraction peak intensity of the PDA-treated materials was higher and more obvious than that of the untreated materials (Fig. 1h;Supplementary Fig. S2), its characteristic crystal peak was at 2 theta 16.79° and 18.55° was assigned to polymer, 2 theta at 27.69°, 43.49°and 50.71° were attributed to inorganic particle, than that of the untreated materials (Fig. 1h;Supplementary Fig. S2), evidencing that the crystallization peak of PLCL after PDA modification and BG addition was further enhanced. Because of the plentiful groups of PDA, it might provide a synergistic effect on BG to promote the crystallization of PLCL. The surface topography of PLCL/PDA and PLCL/BG2/PDA became rough after PDA coating (Fig. 2f;Supplementary Fig. S3). It showed the distribution of BG and PDA in the scaffold and different elements in different groups. We can see that the surface of PLCL group is smooth, and BG is evenly distributed on the surface of PLCL/BG scaffold. After PDA treatment, the surfaces of the two groups of scaffolds became rough, and the active substances of PDA were evenly distributed. And elements contained in the composite scaffolds have been detected. This finding indicates that it contributed to cell adhesion and spreading, which is consistent with references [36, 47].

The surface hydrophilicity and hydrophobicity of the PLCL and PLCL/BG2 scaffolds were evaluated by dynamic contact angle analysis. Compared with the PLCL group, the contact angle of the PLCL/BG2 group increased in a statistical model. This is due to the enrichment of BG on the surface of the material as the content of BG increased, resulting in an increase in the hydrophobicity of the composite scaffold [47]. High hydrophilicity favors cell adhesion and proliferation [33]. However, the hydrophilicity of the PLCL and PLCL/BG2 composite scaffolds increased after PDA treatment (Fig. 2g). When the measurement time was prolonged, the contact angle value decreased slightly, due to the effects of gravity on the water droplets.

### In vitro cell enrichment and osteogenic activity of the porous scaffolds

**Figure 3.**
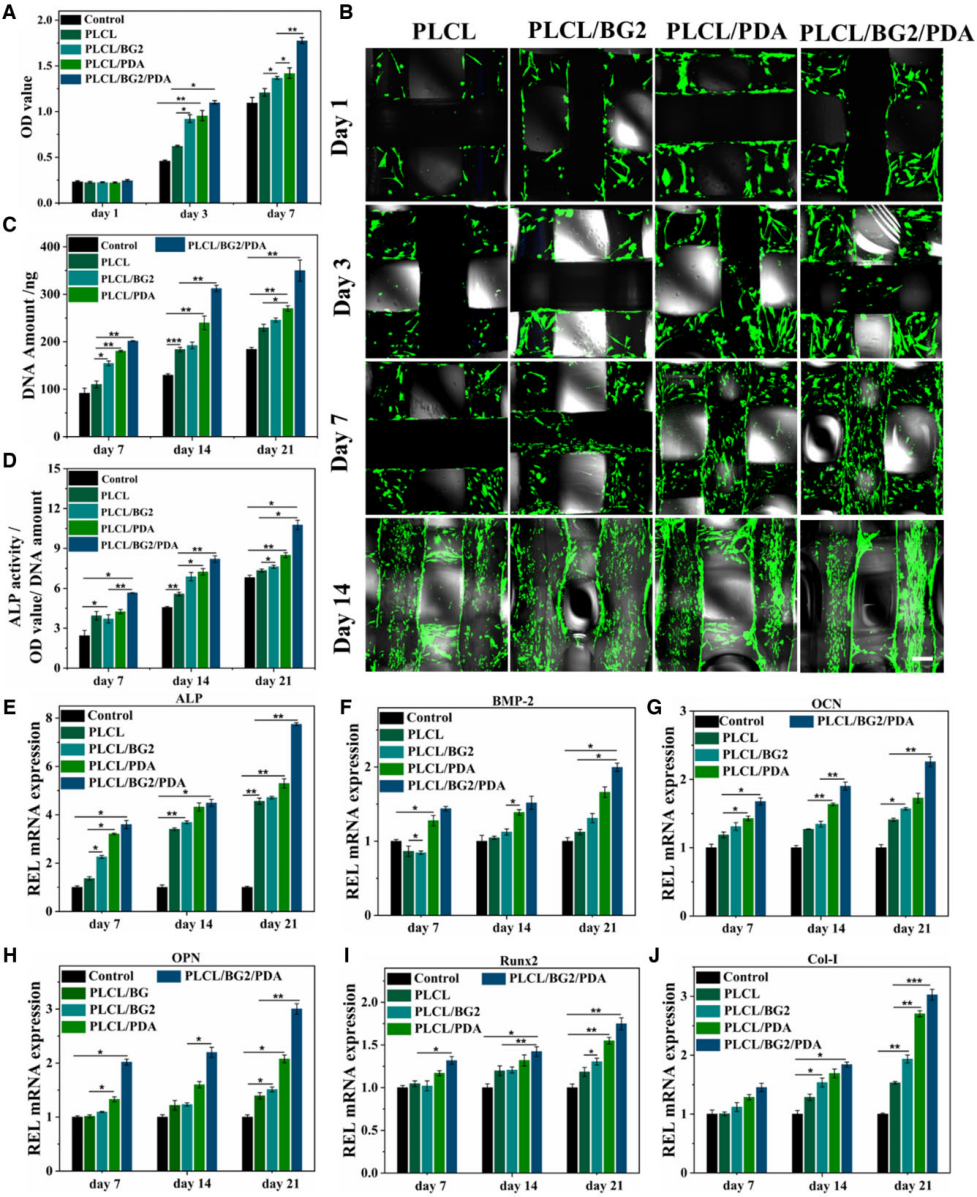
*In vitro* osteogenic proliferation and differentiation of PDA-coated PLCL/BG composite scaffolds. (**A**) The cell proliferation of hBMSCs cultured with four porous scaffolds for different time points. (**B**) Fluorescent images of hBMSCs at four different time points after seeding on PLCL and PLCL/BG before and after PDA modification. Scale bars, 200 μm. (**C**) PicoGreen assay of the DNA amount in hBMSCs cultured with four scaffolds on Days 7, 14 and 21 and (**D**) quantitative analysis of the relative ALP activity of hBMSCs cultured with four scaffolds on Days 7, 14 and 21. (**E**–**J**) Osteogenic differentiation-related gene expression of hBMSCs, including ALP, BMP2, OCN, OPN Runx2 and Col-I, after incubation for 7, 14 and 21 days (*P < 0.05, **P < 0.01 and ***P < 0.001).

To study the viability, proliferation and differentiation of cells on scaffolds, *in vitro* biological experiments of 3D-printed PLCL, PLCL/BG2, PLCL/PDA and PLCL/BG2/PDA scaffolds were conducted using hBMSCs (Fig. 3a). The results demonstrate that the cell proliferation of the scaffolds in these four groups showed a similar trend after co-incubating 3 and 7 days, and the OD value of various scaffolds gradually increased at different times. Compared to the PLCL and PLCL/BG2 scaffolds, many more cells were found in the PLCL/PDA and PLCL/BG2/PDA scaffolds after different times of *in vitro* coculture. In addition, an obvious difference in cell proliferation was found between the PLCL/BG scaffold and the PLCL/BG2/PDA scaffold after 7 days of culture. The above results indicate that the 3D-printed scaffolds had no obvious cytotoxicity. Because of cells of every group have short wall sticking time after culturing 1 day, change of OD value was not obvious of all group. To evaluate the natural bone-reviving capability of various scaffolds, hBMSCs were cultured with various scaffolds (Fig. 3b). Laser scanning confocal microscope measurements showed that after 1, 3, 7 and 14 days of *in vitro* coculture, the hBMSCs adhered and sprawled well on the surface of all scaffolds. In addition, many live hBMSCs were observed in the porous scaffolds with BG-blended and PDA-treated surfaces compared to the PLCL scaffold. Both the polydopamine coating and BG could promote cell proliferation and facilitate cell adhesion by significantly increasing the hydrophilicity and microscopic roughness of the PLCL/BG/PDA porous scaffolds. Supplementary Figure S4 shows 3D images of hBMSCs co-incubated with porous scaffolds for 1, 3, 7 and 14 days. The result is the same as that in Fig. 3b. hBMSCs not only grew on the surface of the scaffolds but also proliferated and grew deep into the holes in the scaffold along the walls, especially in the PLCL/BG2/PDA group.

Further cell proliferation and osteoblast differentiation studies (Fig. 3c and d) of hBMSCs cultured with porous scaffolds revealed that the adhesion of cells was relatively better on PLCL/BG2/PDA and PLCL/PDA due to the higher wettability of the scaffold. Compared with the other scaffolds, the PLCL/BG2/PDA group had the highest DNA amount (Fig. 3c). ALP is commonly considered the primary marker of early bone formation [4, 42], and plays an important role in bone mineralization by stimulating pyrophosphate [3]. Subsequently, the osteogenic differentiation of hBMSCs co-incubated with different scaffolds was measured using an ALP kit. With increasing time, the ALP activity of all the scaffolds was improved. Compared to the pure PLCL scaffold, the results show that the ALP activity of hBMSCs on Days 7, 14 and 21 was remarkably promoted with the BG blending and PDA-ornamented PLCL scaffolds. The above results indicate that bioactive glass and polydopamine could significantly promote the osteogenic induction and osteogenic differentiation of hBMSCs. Subsequently, the osteogenic differentiation of hBMSCs co-incubated with porous scaffolds was measured using AR staining (Supplementary Figs S5 and S6). To investigate osteogenic differentiation in the late stage of osteogenic-related cells, AR is widely used to stain deposited calcium [48, 49]. When hBMSCs were cocultured with various porous scaffolds for 14 and 21 days, the PLCL/BG2/PDA group showed the most calcium mineral nodules.

It has been reported that osteoblast differentiation is a prerequisite for bone regeneration [3, 50], and the osteogenic behavior of different porous structures has been successfully assessed. Thus, RT-PCR was used to observe the expression of the osteogenic-specific genes ALP, OCN, OPN, BMP-2, Runx2 and Col-1 in cells cultured with various scaffolds. It is worth noting that the expression of all genes increased with prolonged coculture time (Fig. 3e–j). It was interesting that at different time points, the PLCL/PDA and PLCL/BG2/PDA groups showed evident expression of all genes mentioned above compared to the PLCL scaffolds. Notably, the ALP expression of the PLCL/BG2/PDA group was the highest, followed by that of the PLCL/BG2 and PLCL/PDA groups, while the lowest expression was observed for PLCL (Fig. 3e). The PLCL/BG2/PDA group had the highest expression level of BMP-2 on the 21st day (Fig. 3f). Figure 3g shows the OCN gene expression of scaffolds in each group. The gene expression of OCN in the PLCL/BG/PDA group reached the maximum value on the 21st day. The expression of the OPN gene shown in Fig. 3h is similar to that of the OCN gene, and the expression peaked on the 21st day. Figure 3i shows the expression of the Runx2 gene in each group of scaffolds. On the 7th and 14th days, the Runx2 gene expression between the PLCL/BG2 and PLCL/PDA groups was not significantly different, and there was no statistical significance. On the 21st day, the expression of Runx2 in the PLCL/BG group was higher than that in the PLCL group and had obvious statistical significance. The expression of the Col-I gene in the PLCL/BG, PLCL/PDA and PLCL/BG/PDA groups was markedly better than that in the PLCL scaffold group (Fig. 3j). Based on the above analysis, it is rational to deduce that the PLCL/BG2/PDA scaffold group had excellent enhanced osteogenic functionality.

**Figure 4.**
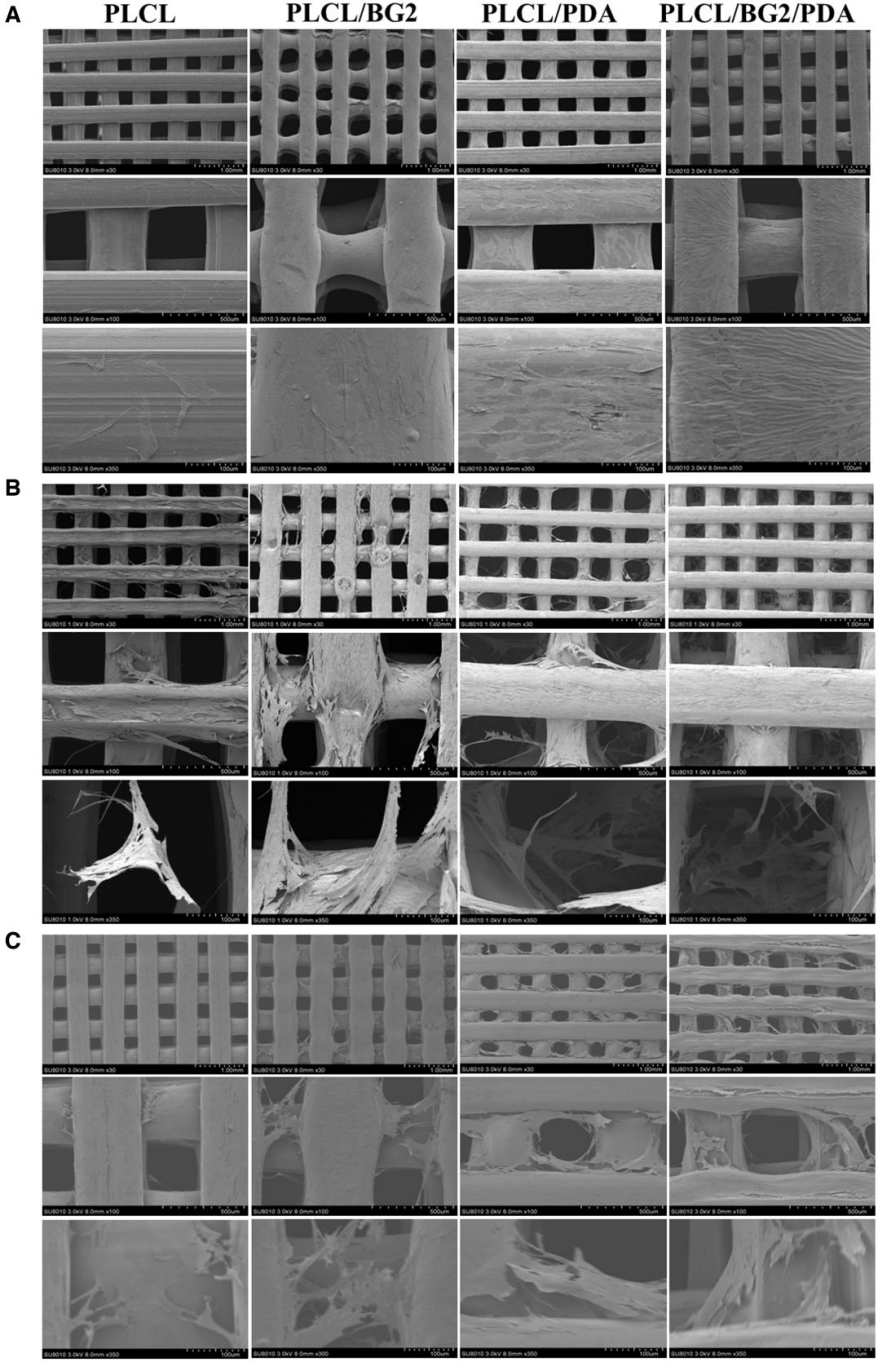
Surface adhesion of hBMSCs cocultured with various scaffolds *in vitro*. (**A**) 3 days, (**B**) 7 days and (**C**) 14 days.

The cell adhesion properties of implants are key factors in promoting osseointegration, so the adhesion properties of porous scaffolds of hBMSCs were also studied. The adhesion morphology of hBMSCs cultured on the scaffold surface for 3, 7 and 14 days was observed by SEM (Fig. 4a–c). It showed that more cells adhered initially to the modified substance, which resulted in the increase of cell viability in the early stage [38], the results were the same as the cell proliferation (Fig. 3a and b). The cells on the scaffolds in each group conglutinated and sprawled well, and hBMSCs grew into the scaffold along the holes. The number of cells on the scaffolds in each group increased with prolonged culture time, and pseudopodia were extended (even across the pores). The PDA coating to an enhanced background adhesion of the modified surface to cells as well as many other biologically active substances [38]. The PLCL/BG2/PDA scaffold had the most adherent cells and a larger spreading area.

### In vivo bone-regenerative capacity of PLCL/BG/PDA scaffolds

**Figure 5.**
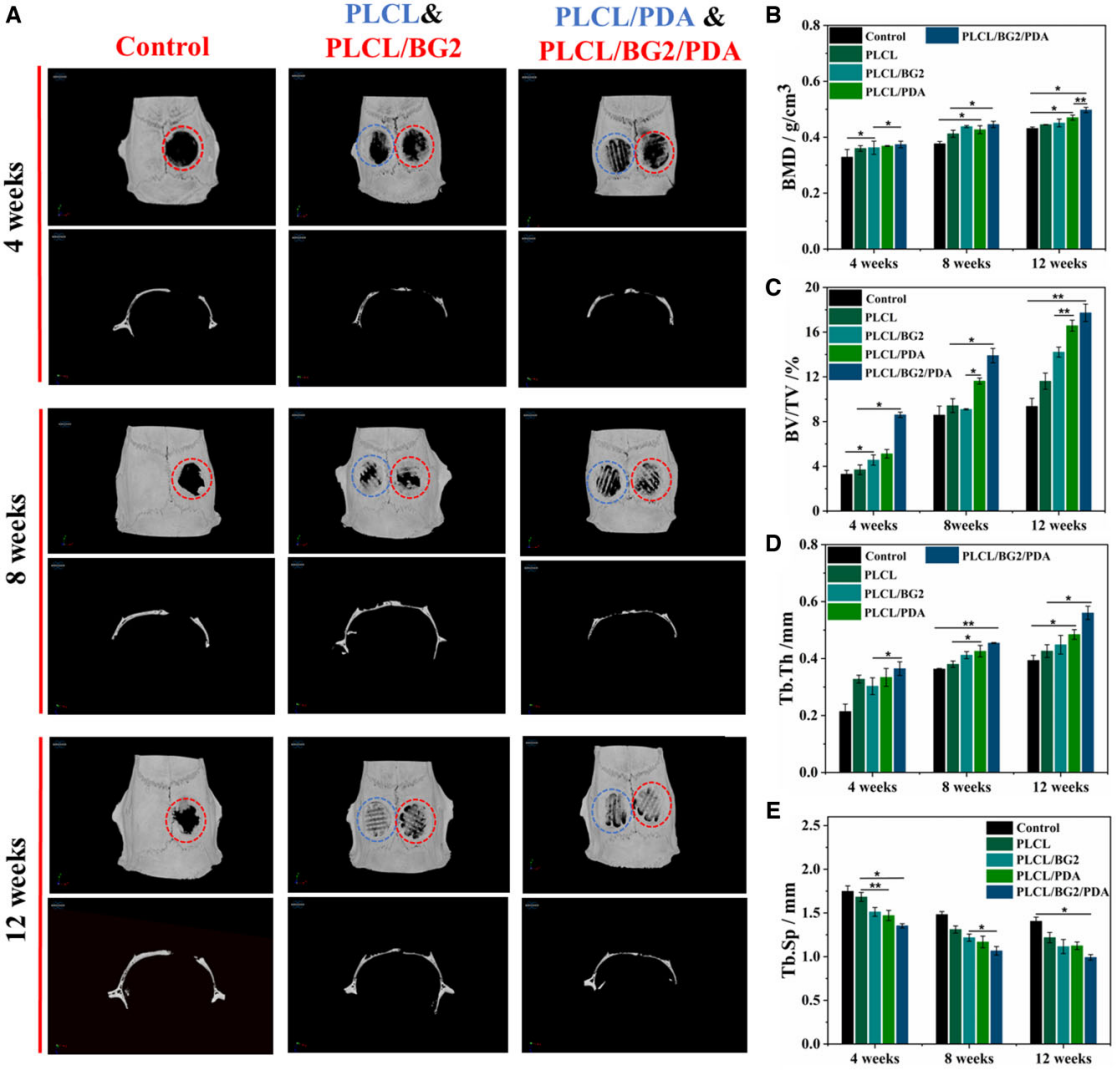
Bone regeneration in a rat calvarial bone defect model. (**A**) Typical 3D reconstruction of micro-CT images. Quantitative analysis of new bone formation in defect areas after implantation of various scaffolds for 4, 8 and 12 weeks: (**B**) bone mineral density (BMD), (**C**) bone volume/total volume (BV/TV), (**D**) trabecular thickness (Tb.Th) and (**E**) trabecular separation (Tb.Sp) (*P < 0.05, **P < 0.01).

A typical SD rat skull defect model was applied to investigate the osteogenic performance of the 3D-printed PLCL/BG2 composite scaffold and PDA-coated PLCL/BG2 scaffold *in vivo*. PLCL, PLCL/BG2, PLCL/PDA and PLCL/BG2/PDA scaffolds were implanted for 4, 8 and 12 weeks, respectively. In different implant cycles, all sterilized scaffolds implanted in the skull defect could degrade and induce different degrees of new bone formation without obvious displacement (Fig. 5a). From the 3D reconstruction, the results of the 4-week specimens show that there was only slight new bone tissue ingrowth at the edges of the four groups of scaffolds, and there was less new bone tissue in the middle of the scaffolds. When the scaffolds were implanted for 8 weeks, the ingrowth of new bone tissue in the inner position of the scaffold increased in all four groups, and the bone mass of the PLCL/BG2/PDA group was significantly higher than that of the other groups.

After 12 weeks of implantation, a large amount of new bone was found in the PLCL/BG2/PDA groups, and the new bone tissue in the PLCL/BG2/PDA group basically covered the entire defect area and extended to the center of the defect, forming a complete bone structure without significant scaffold fracture and collapse, which was better than that in the other groups in terms of osseointegration. It was speculated that the more abundant blood supply and nutrient transmission on the side of the dura mater can promote bone regeneration. The PLCL group also had obvious bone regeneration, the new bone formed extended very little to the center of the defect area, and the regenerated bone tissue was incomplete. The control blank group retained a larger defect area and regeneration did not extend to the center of the defect cavity. However, they were filled with a great amount of fibrotic tissue.

To quantitatively analyze the repair effect of scaffold materials on bone defects, CTAn software was used to analyze the new formation bone-related parameters, including the BV/TV, BMD, Tb.Th and Tb.Sp in rat calvarial defects (Fig. 5b–e). Compared to the PLCL scaffold alone, the quantitative analysis showed higher BMD and BV/TV in the PLCL/BG2/PDA scaffold group at different cycles (Fig. 5b and c), suggesting that the PLCL/BG2/PDA scaffold significantly promoted more mineralization than the PLCL scaffold. Tb.Sp and Tb.Th are essential for bone regeneration assessment because trabecular bone forms a 3D network with an irregular structure within the marrow cavity and assists hematopoiesis [51]. As shown in Fig. 5d–e, the PLCL/BG2/PDA group showed the minimum Tb.Sp and maximum Tb.Th values. References have been reported [3, 52, 53], and an increase in Tb.Sp or decrease in Tb.Th demonstrate that bone catabolism is more important than bone anabolism [3]. PDA decoration might provide an active effect on cell differentiation and adhesion, and BG sustained release of bioactive ions (Ca, Si, P) of 3D-printed PLCL/BG2/PDA porous scaffolds might enhance surface bioactivity, which ultimately leads to better osteogenesis.

**Figure 6.**
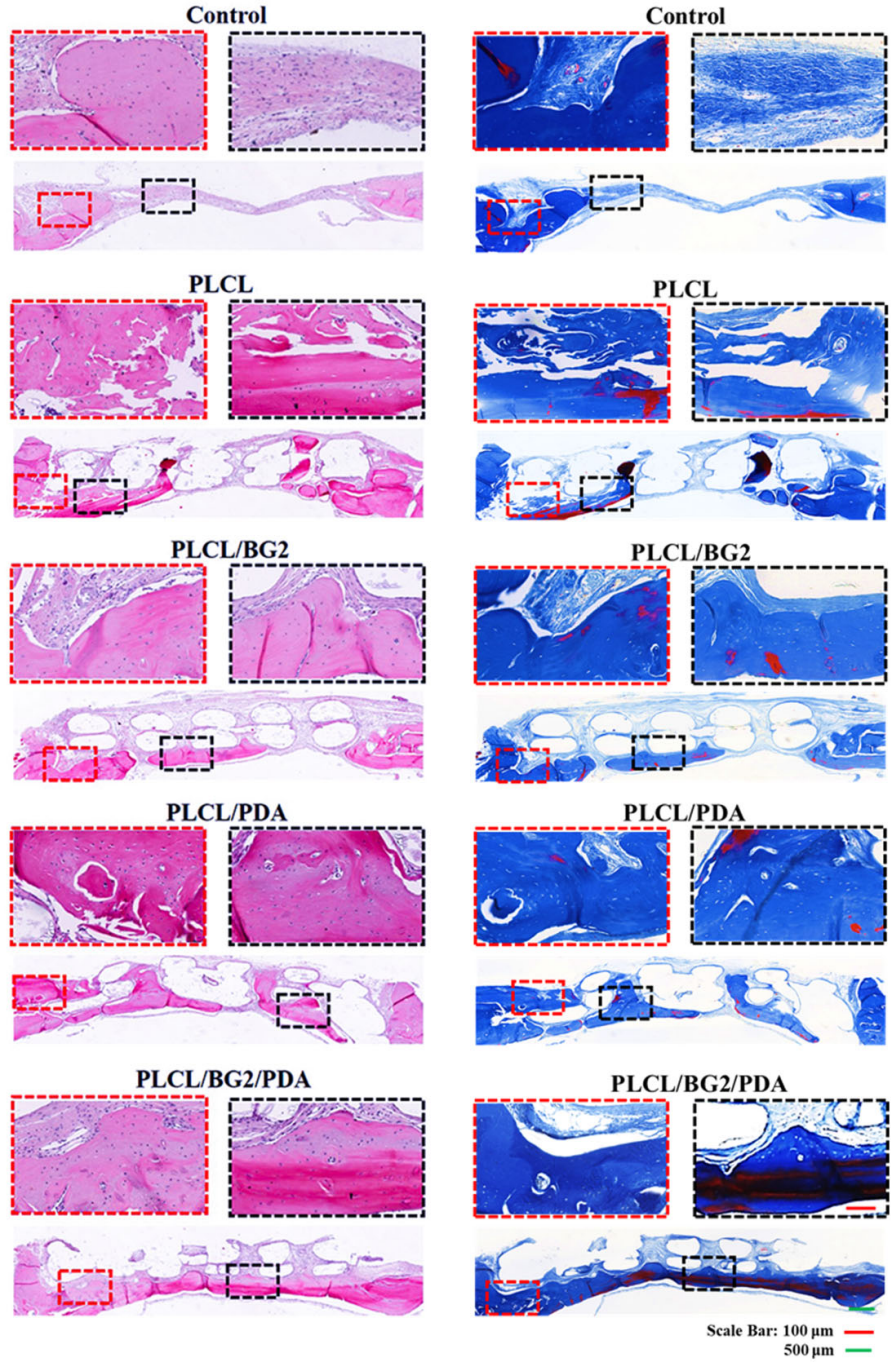
*In vivo* bone regeneration. H&E staining and MT staining of SD rat skulls with cranial defects after implantation of various scaffolds for 12 weeks.

Figure 6 and Supplementary Figs S7 and S8 show the H&E staining of the five defect groups after decalcification and paraffin sectioning. At the fourth week of implantation into the defect, no new bone formation was observed, but massive fibrotic tissue was observed in the blank group (Supplementary Fig. S7). After 8 weeks, a few new bone tissues were found in the control group (Supplementary Fig. S8). However, the regeneration bone of the PLCL/BG2, PLCL/PDA and PLCL/BG2/PDA groups could be seen after the first 4 weeks (Supplementary Fig. S7), and the new bone was increased by degrees over 8 and 12 weeks (Supplementary Fig. S8). More importantly, it was obvious that the newly formed bone grew toward the interior of all the porous scaffolds, demonstrating that the porous structure containing BG showed fairly satisfactory osteogenic properties. The PLCL/BG2/PDA group had the most regeneration of new bone tissue; at the end of 12 weeks, a large amount of new bone tissue was formed, even inside the inner scaffold. New regenerated bone fully filled the bone defect area, and the newly generated bone tissue tightly integrated with the original tissue (Fig. 6). In addition, Figs 5a and 6 indirectly reflect that not only the PLCL/BG/PDA scaffold, but also the other scaffold groups had not degraded after 12 weeks of implantation. We conjectured that PDA has small effect of the degradation property on the PLCL and PLCL/BG scaffold. But the sodium hydroxide treatment might aggravate degradation of PLCL/PDA scaffolds and PLCL/BG/PDA scaffolds (Supplementary Fig. S9) [54]. Much more deeply and comprehensively work needs to be studied on the degradation performance of scaffolds *in vitro* and *in vivo* in the future.

MT staining was also used to test the bone regeneration capability of the porous scaffold. The results of the MT staining experiment are the same as the H&E staining results (Fig. 6; Supplementary Figs S7 and S8). For the control group, only some new bone was regenerated at the edge of the defect area, even at 12 weeks (Fig. 6), while the four porous scaffold groups had many blue-stained areas that switched to red, especially the PLCL/BG2/PDA group, indicating that new mature bone was formed in the center of the defect area. In the blank control group, there was a large amount of fibrous tissue in the defect, and the Masson staining was almost blue. The results of the above two staining experiments are consistent with the micro-CT analysis results. We speculate that a possible reason for the wonderful new bone formation capability of ternary porous scaffolds is the sustained release of bioactive multifunctional ions (Ca, Si, P) from BG, which have been commonly studied to promote bone repair and healing [10, 27, 29]. Si might promote bone regeneration by the AMPK/ERK1/2 signaling pathway [4].

**Figure 7.**
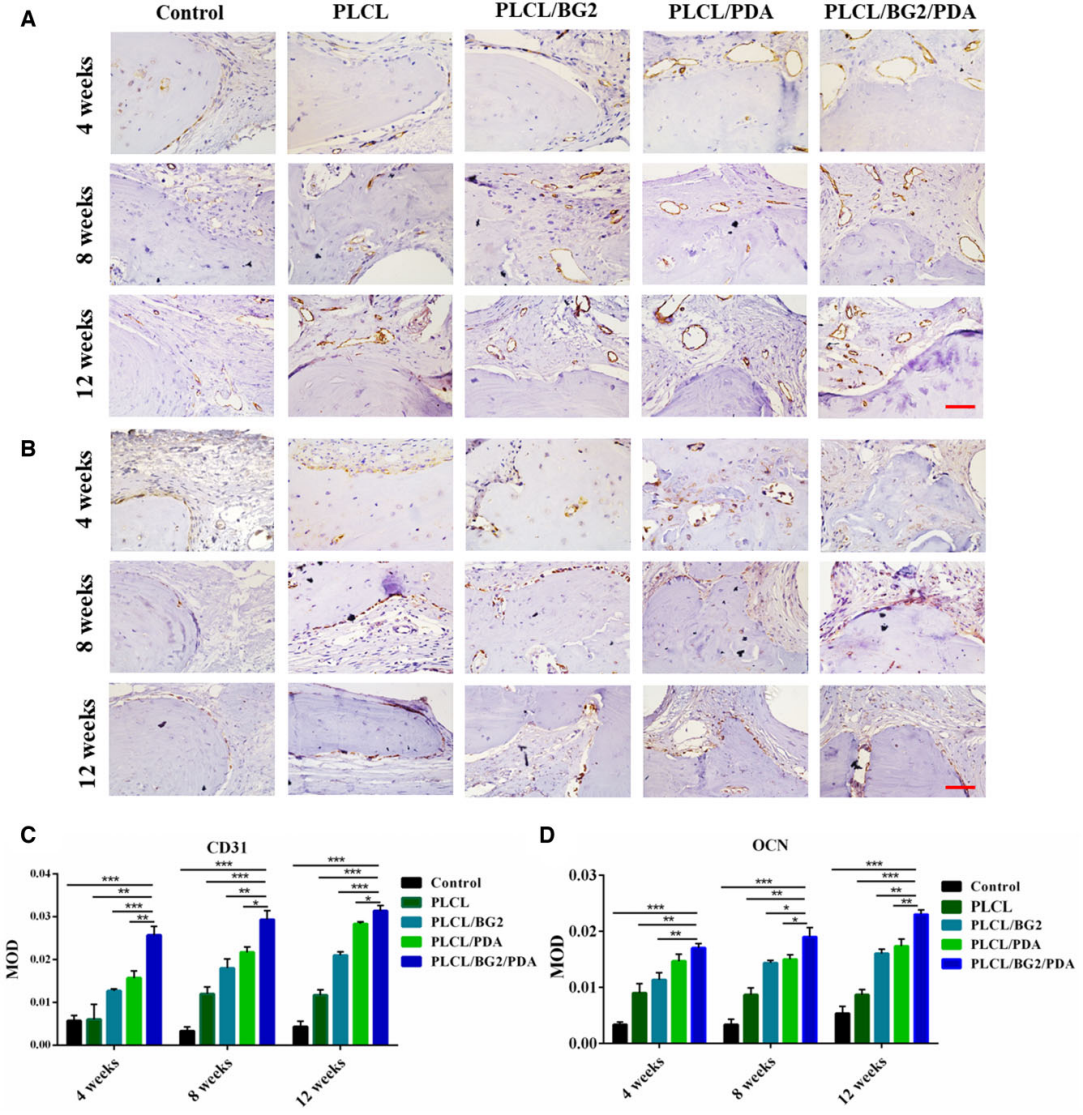
IHC staining of (**A**) CD31 and (**B**) OCN at 4, 8 and 12 weeks. Scale bars, 100 μm. Semi-quantitative analysis for the CD31 (**C**) and OCN (**D**) at 4, 8 and 12 weeks (*P < 0.05, **P < 0.01).

IHC staining was used to validate the presence of new bone regeneration in the defect area. To study osteogenesis-related expression during bone regeneration, IHC staining of the associated bone protein and vascular-associated protein (OCN, CD31) expression analysis were conducted (Fig. 7a and b). The hematoxylin-stained nuclei were blue, and double antibody (DAB) staining was brown. The relative expression levels of OCN and CD31 in the PLCL/BG2/PDA scaffold were significantly distinct from those in the other three scaffold groups after implantation for different cycles, and PLCL/BG2/PDA was verified to have the best angiogenesis and osteogenesis. The defect area in the control group was mainly composed of fibrous tissue, with very little positive expression of OCN and CD31. Compared with other groups, after 12 weeks of implantation, the newly generated bone in the PLCL/BG2/PDA group was much denser and richer and had numerous blood vessels.

The results are consistent with the analysis of H&E and MT staining. CD31 and OCN positive areas and mean optical density of the PDA groups were significantly increased compared with 4 and 8 weeks (Fig. 7c and d). The PLCL/BG/PDA group showed the best positive expression. This may be attributed to the improved hydrophilicity of the scaffold due to polydopamine, the release of bioactive particles such as Ca, Si and P in the bioactive glass and the interconnected inner structure, thus PLCL/BG2/PDA exhibits excellent bone regeneration ability.

## Conclusion

In summary, we successfully constructed a 3D-printed PLCL/BG scaffold with PDA decoration to achieve valid bone repair and regeneration in a rat cranium defect model. The released bioactive ions (Ca, Si and P) from BG could accelerate the osteogenic proliferation and differentiation of hBMSCs *in vitro* and promote new angiogenesis formation *in vivo.* The 3D porous composite structure fully adopted the functional properties of PLCL, bioceramics and PDA. Natural bone tightly integrated with the implant, and new bone ingrowth toward the center of the scaffold accelerated the fast and lasting osseointegration of the PLCL/BG/PDA porous structure. In general, post-treatment of PLCL/BG2 composite scaffolds with PDA modification offers better possibilities for the evolution of bioactivity connected customized porous scaffolds and various growth factor/organic/inorganic composite implants to achieve bone defect repair in the future.

## Supplementary Material

rbad062_Supplementary_Data
