# Supplementary material for: 3D-printed porous functional composite scaffolds with polydopamine decoration for bone regeneration
Source: Regen Biomater. 2023 Jun 21;10:rbad062. doi: 10.1093/rb/rbad062 (PMC10374492; doi:10.1093/rb/rbad062)
Supplement: rbad062_Supplementary_Data [file rbad062_supplementary_data.docx]

Supporting Information

**3D Printed Porous Functional Composite Scaffolds with Polydopamine Decoration for Bone Regeneration**

Jin Qi^1, 2, 3^, Yili Wang^1, 2^, Liping Chen^1, 2^, Linjie Chen^4^, Feng Wen^1, 2^, Lijiang Huang^5^, Pfukwa Rueben ^6^, Chunwu Zhang^1,^* and Huaqiong Li^1, 2,^ *

^1^ Joint Centre of Translational Medicine, Department of Orthopaedics, The First Affiliated Hospital of Wenzhou Medical University, Wenzhou, Zhejiang 325035, P. R. China.

^2^ Joint Centre of Translational Medicine, Zhejiang Engineering Research Center for Tissue Repair Materials, Wenzhou Institute, University of Chinese Academy of Sciences, Wenzhou, Zhejiang Province, 325011, P. R. China.

^3^ University of Chinese Academy of Sciences, Beijing, 100049, P.R. China.

^4^ The Second Affiliated Hospital of Wenzhou Medical University, Wenzhou, Zhejiang Province, 325035, P. R. China.

^5^ The Affiliated Xiangshan Hospital of Wenzhou Medical University, Ningbo, Zhejiang 315700, P.R. China.

^6^ Department of Chemistry and Polymer Science, Stellenbosch University, Matieland, Stellenbosch 7602, South Africa.

* Correspondence: Huaqiong Li, [lihq@ucas.ac.cn](mailto:lihq@ucas.ac.cn); Chunwu Zhang, [zcw6681@126.com](mailto:zcw6681@126.com).

**Table S1** Thermal property of PLCL/BG composite scaffolds. a) first heat. b) second heat.

**a**

| Samples | Tg/℃ | Tm/℃ | △Hm/J/g |
| --- | --- | --- | --- |
| PLCL | 32.40 | 155.65 | 34.8445 |
| PLCL/BG2wt% | 45.10 | 156.636 | 28.6012 |
| PLCL/BG5wt% | 23.41 | 158.13 | 24.7935 |
| PLCL/BG8wt% | 32.86 | 159.49 | 30.1575 |

**b**

| Samples | Tg/℃ | Tm/℃ | △Hm/J/g |
| --- | --- | --- | --- |
| PLCL | 41.15 | 154.64 | 4.1874 |
| PLCL/BG2wt% | 41.89 | 153.63 | 0.5746 |
| PLCL/BG5wt% | 38.14 | 153.45 | 8.0005 |
| PLCL/BG8wt% | 32.67 | 141.15 | 2.1282 |

**Table S2.** Decomposition temperature and weight loss of composite scaffolds

| **Samples** | **Td (℃)** | **Weight loss (%)** |
| --- | --- | --- |
| PLCL | 313.97 | 99.079 |
| PLCL/BG2wt% | 304.65 | 95.781 |
| PLCL/BG5wt% | 275.66 | 93.037 |
| PLCL/BG8wt% | 265.57 | 86.560 |


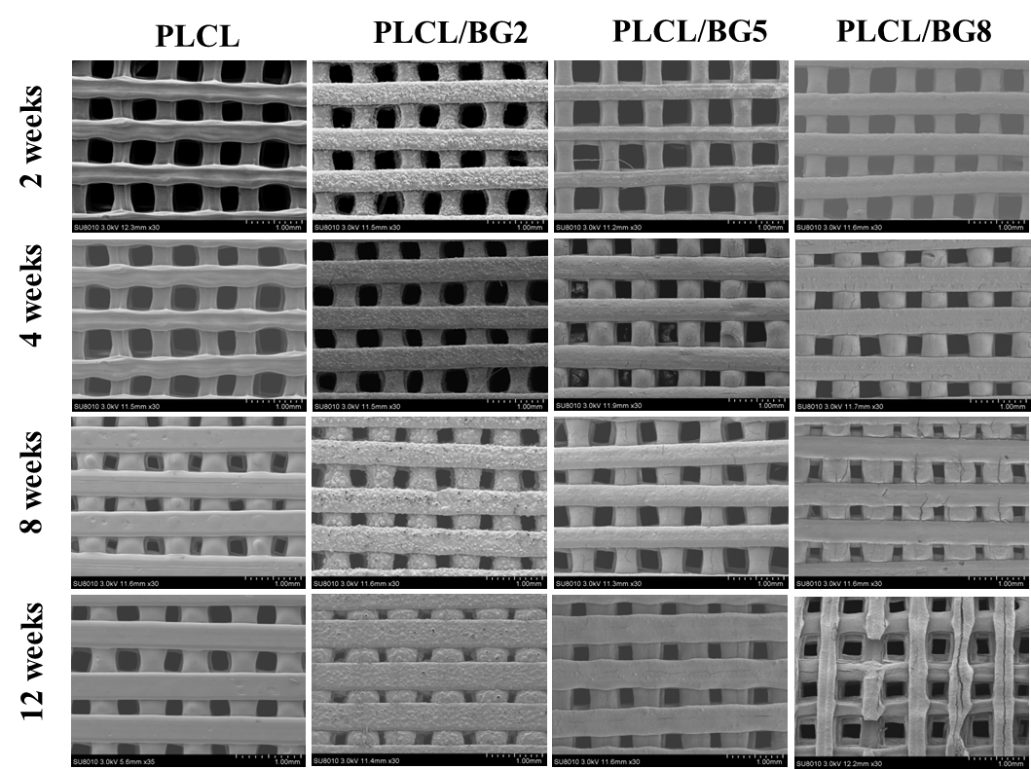


**Fig. S1.** Micromorphology of PLCL/BG composite scaffolds after degradation different cycles in vitro.


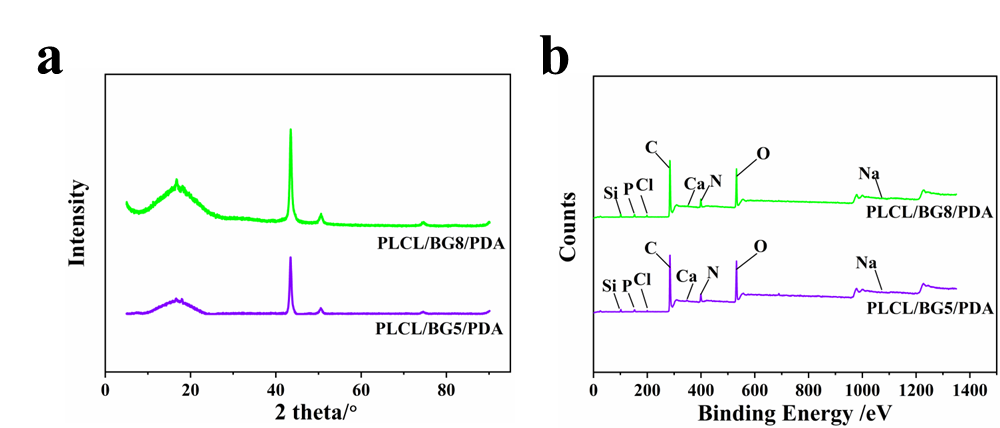


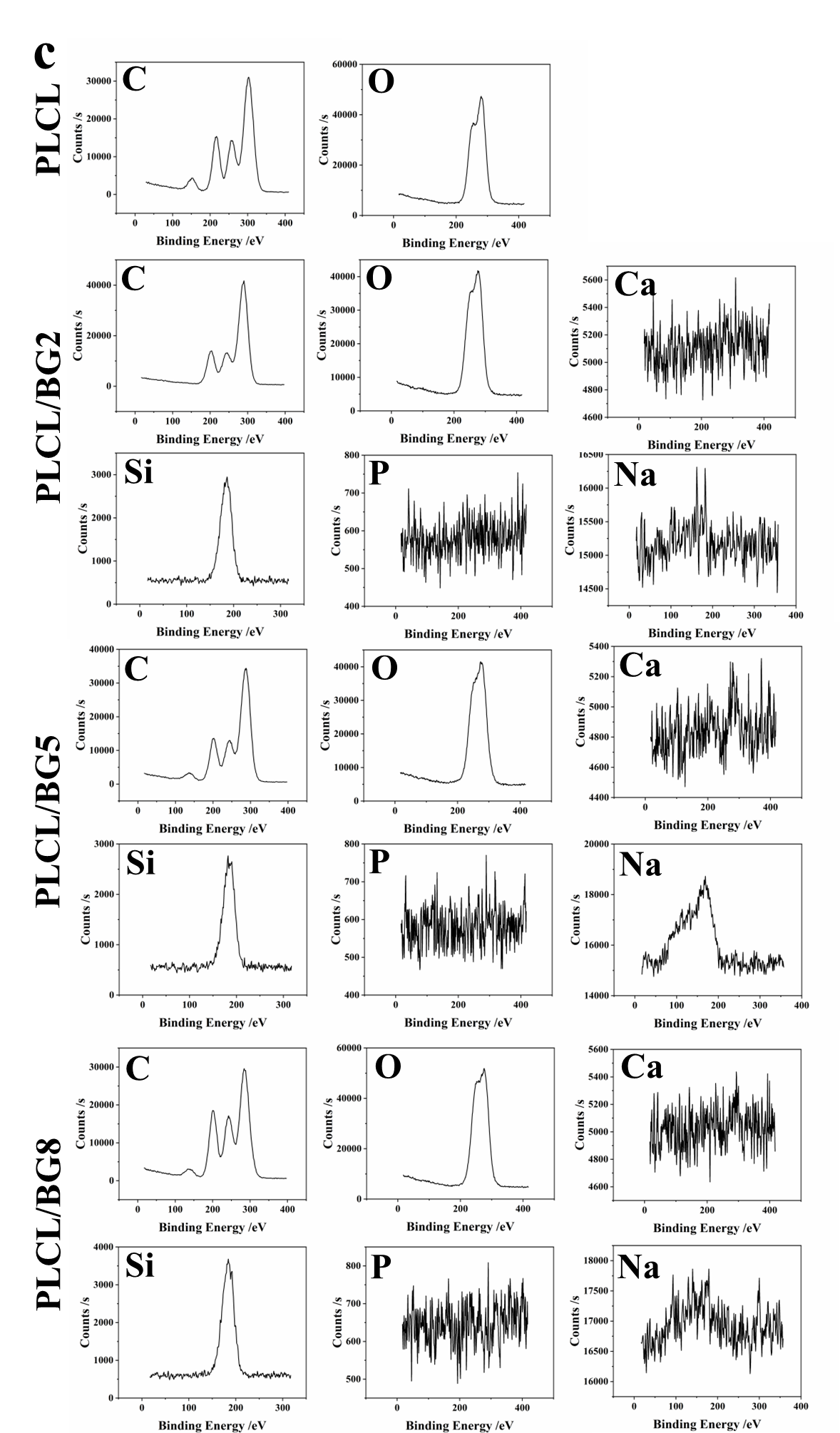


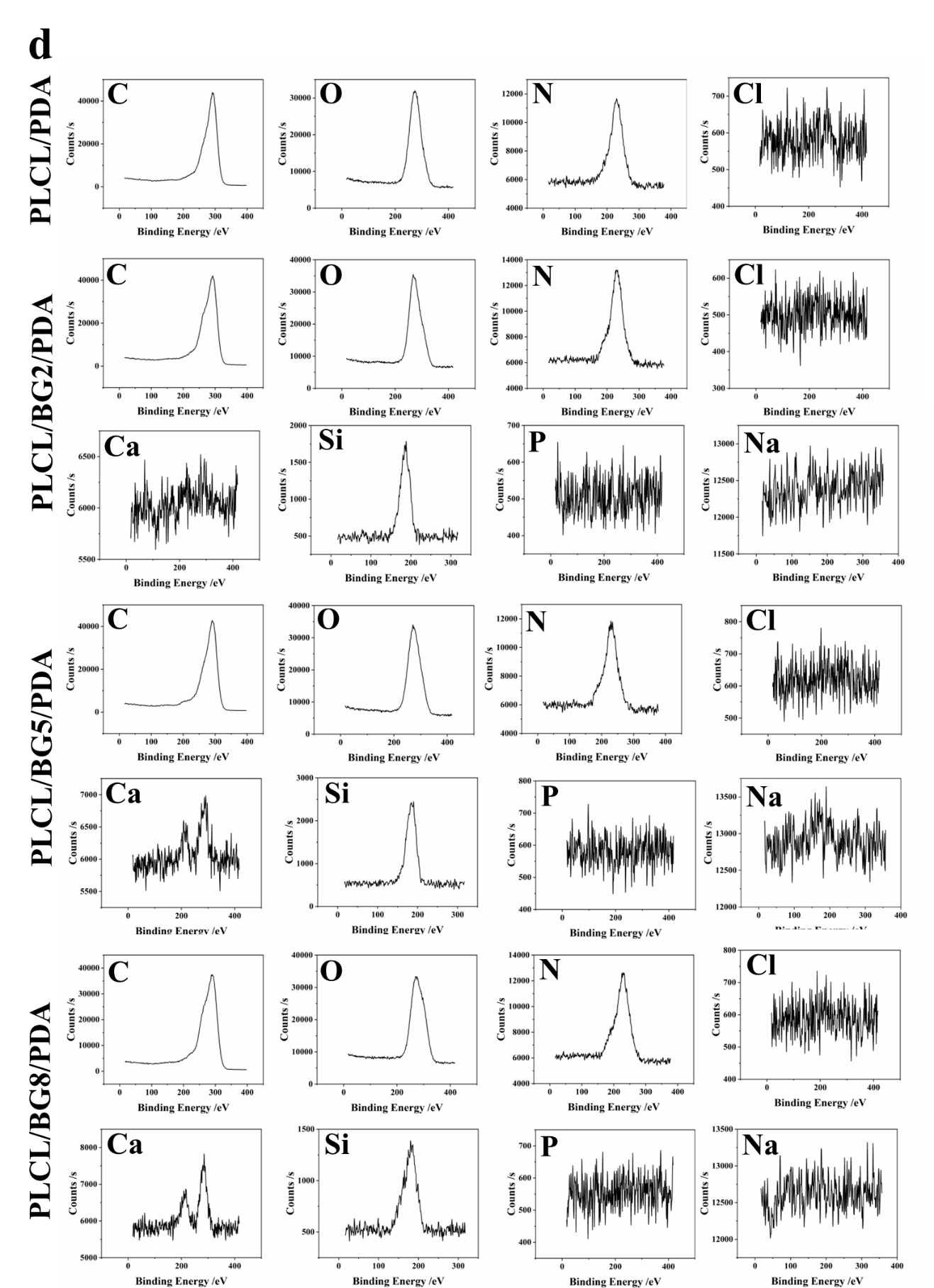


**Fig. S2.** X-Ray Diffraction (a) and X-ray Photoelectron Spectroscopy (b) of 3D printed PLCL/BG composite scaffolds after PDA decorating. The magnified images of all elements of PLCL and PLCL/BG composites before (c) and after PDA coating (d).


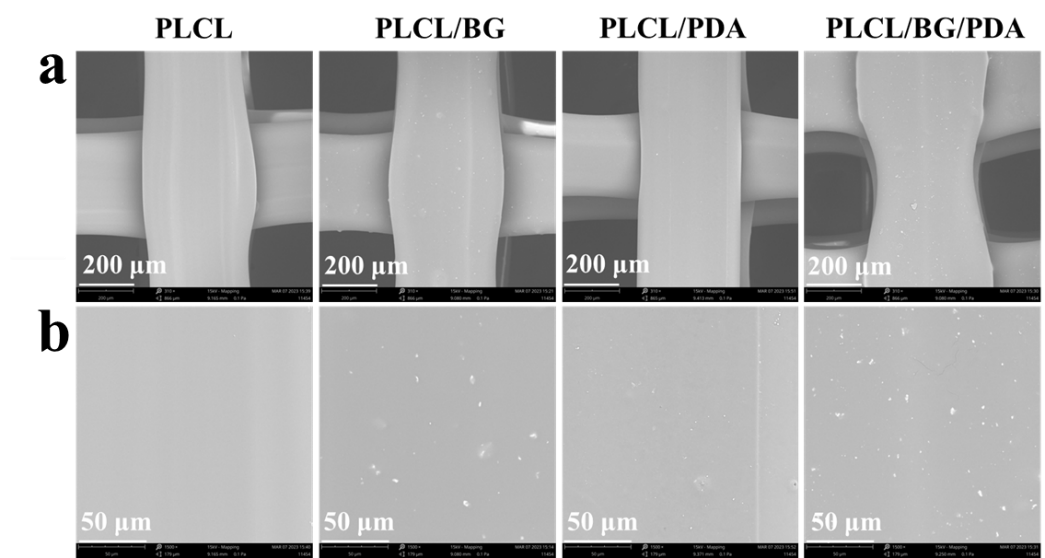


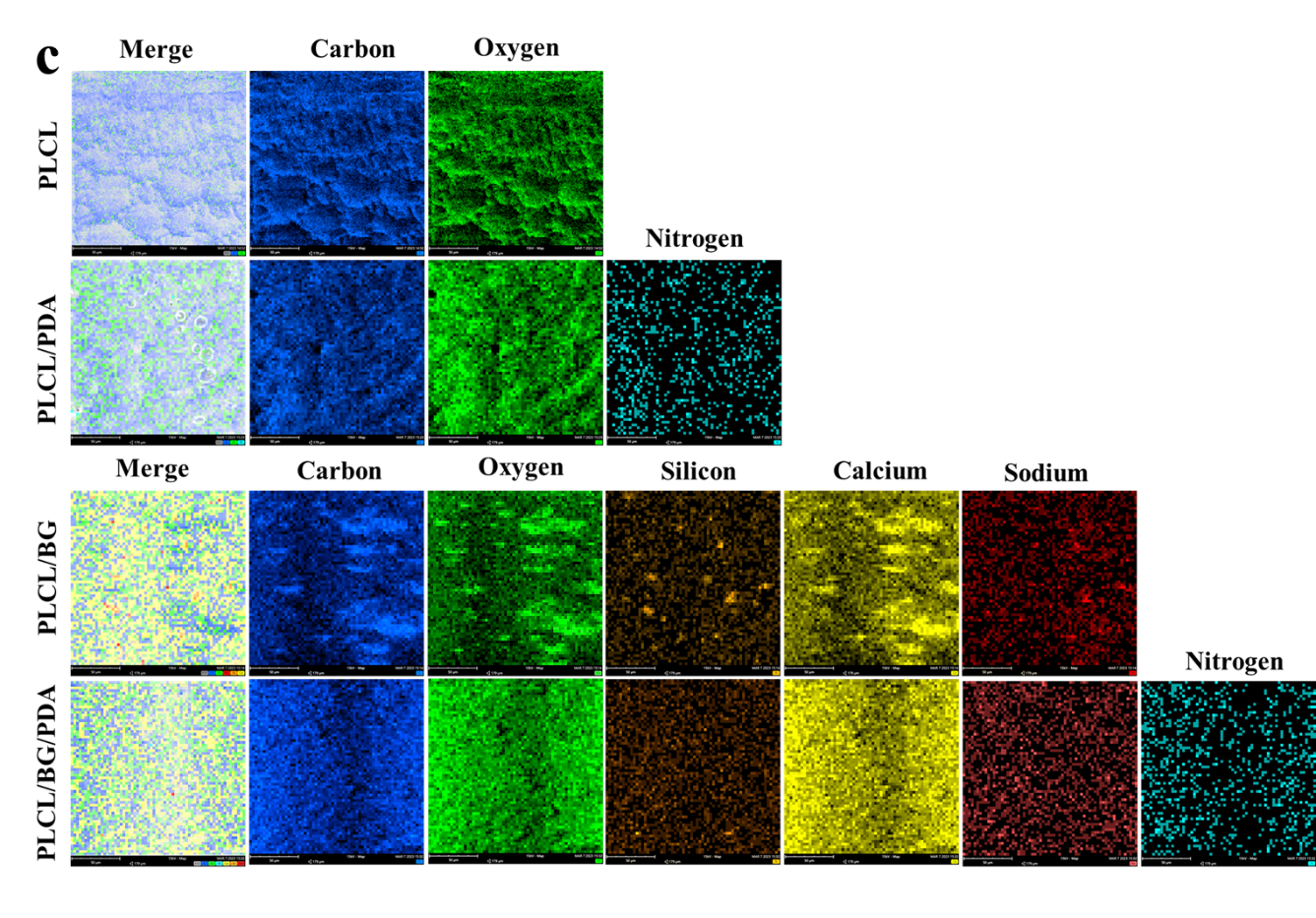


**Fig. S3.** SEM and elements distribution images of PLCL and PLCL/BG2 before and after PDA coating. (a) SEM images of scaffolds 310X and (b) SEM images of scaffolds 1500X, (c) Elements distribution images.


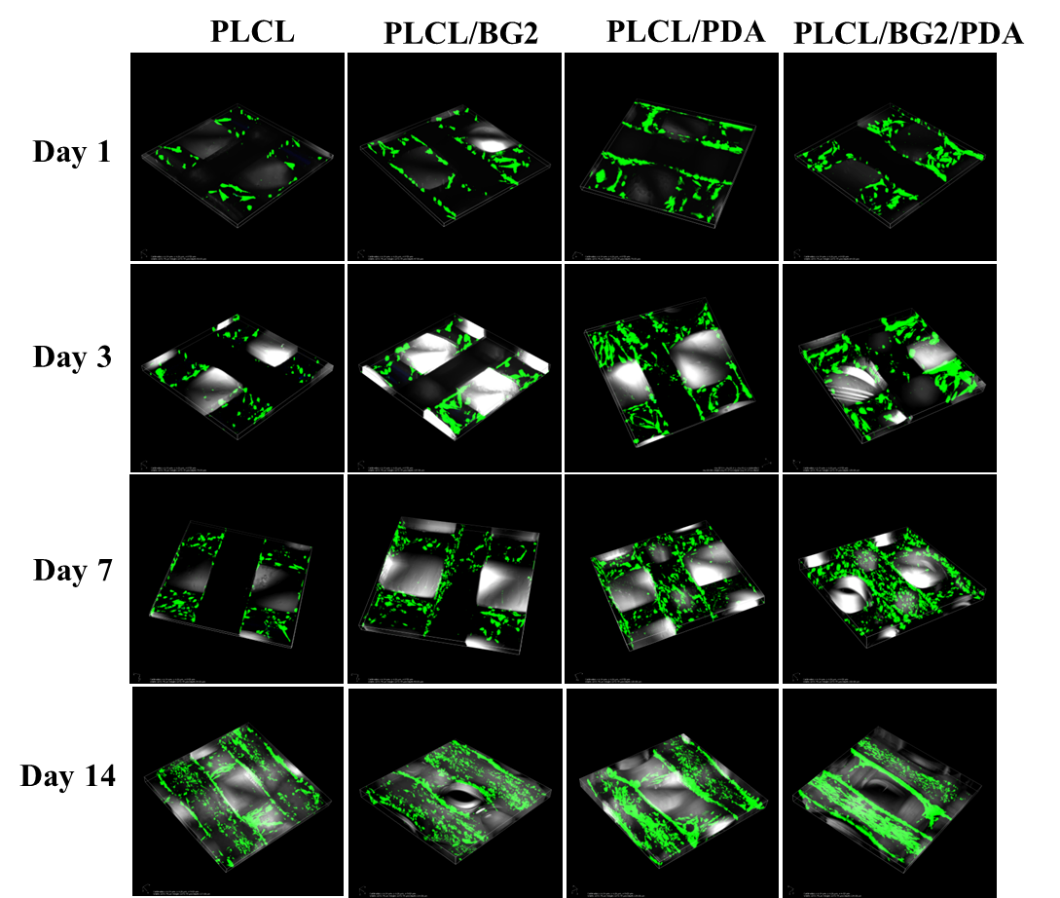


**Fig. S4.** Three-dimensional images of hBMSCs co-cultured with various scaffolds for 1, 3, 7 and 14 days.


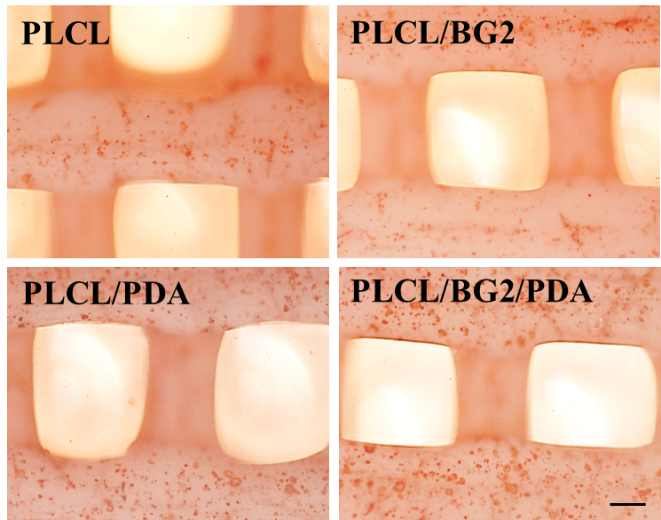


**Fig. S5.** Representative images of alizarin red (AR) staining for hBMSCs cultured with porous scaffold on 14 days. Scale bar, 100 μm.


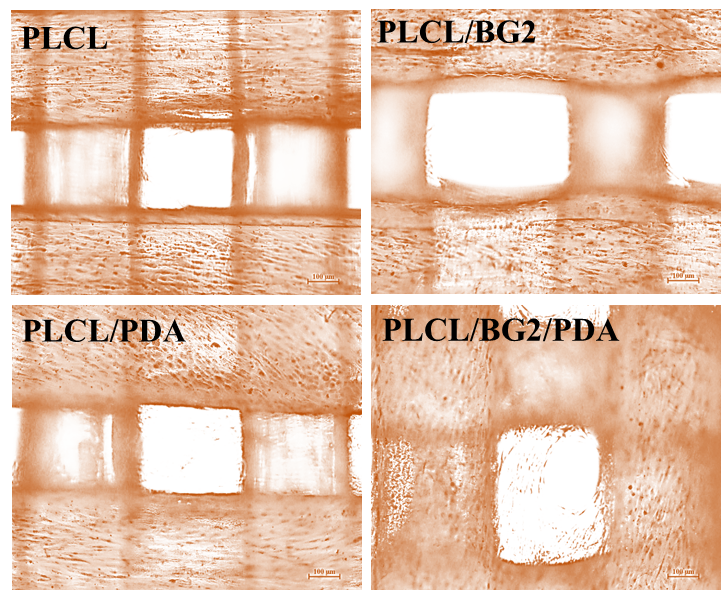


**Fig. S6.** Representative images of alizarin red (AR) staining for hBMSCs cultured with porous scaffold on 21 days. Scale bar, 100 μm.


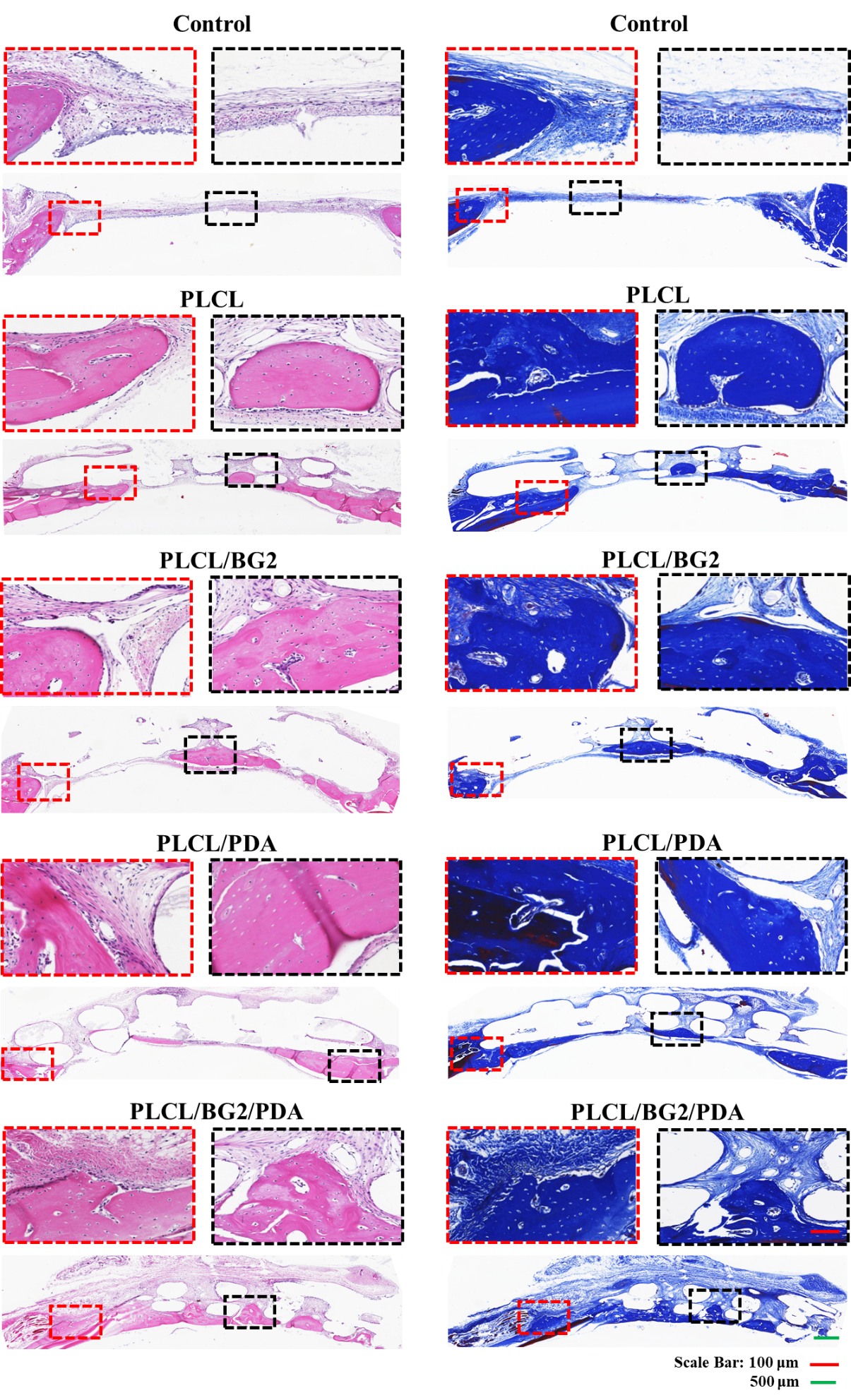


**Fig. S7.** In vivo bone regeneration. H&E staining and Masson’s trichrome staining of the craniums with cranial defects after implantation of various scaffolds for 4 weeks.


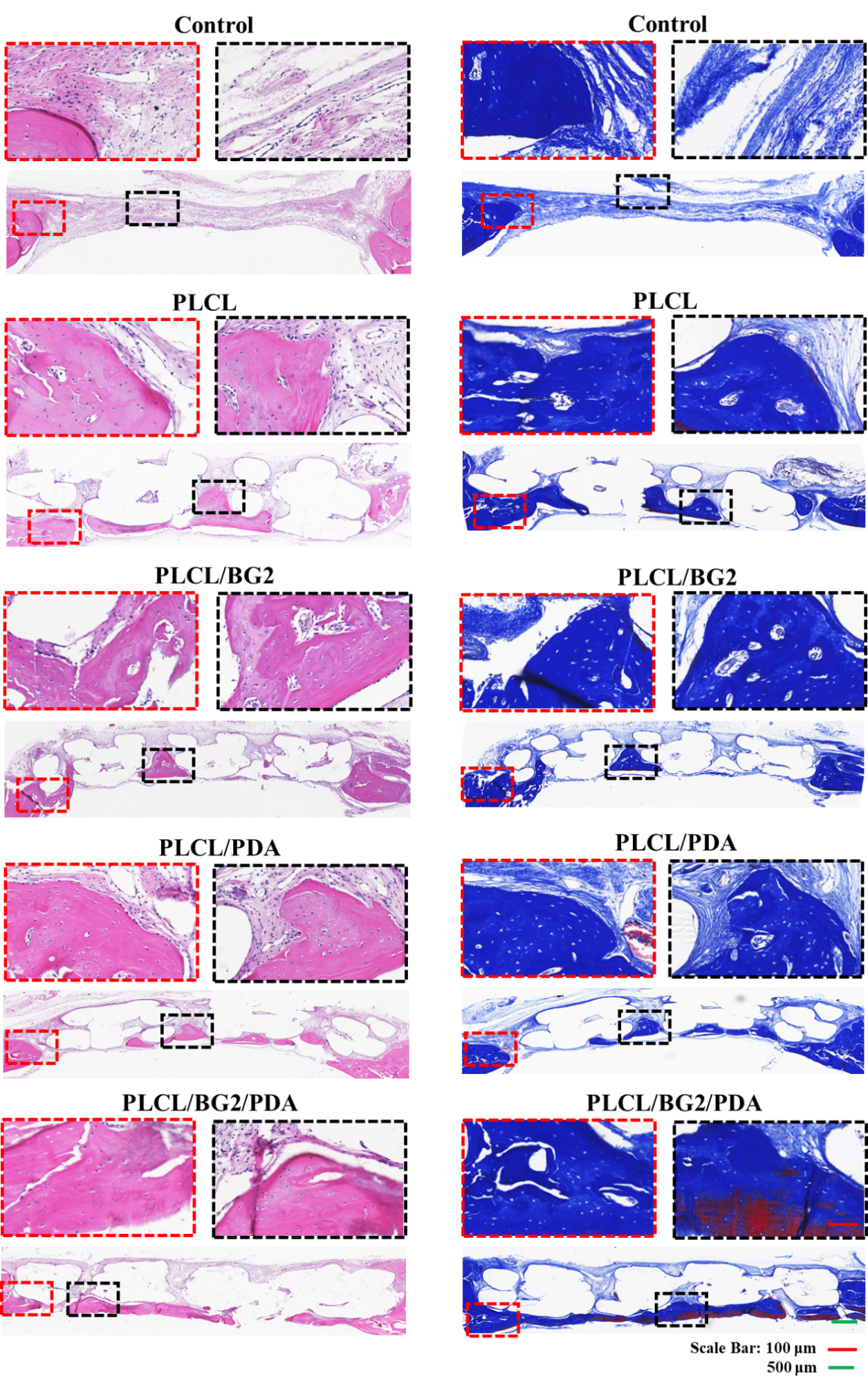


**Fig. S8.** In vivo bone regeneration. H&E staining and Masson’s trichrome staining of the craniums with cranial defects after implantation of various scaffolds for 8 weeks.

**Accelerate Degradation of PLCL/PDA scaffolds and PLCL/BG2/PDA scaffolds**

In addition, we conducted a preliminary experiment to accelerate degradation of PDA coating scaffolds. Firstly, we prepared the PLCL/PDA and PLCL/BG2/PDA scaffold. According to the reference (Evaluation of polycaprolactone scaffold degradation for6 months in vitro and in vivo. doi:[10.1002/jbm.a.32052](https://www.x-mol.com/paperRedirect/1212950078866399243)), degradation properties of PDA coating scaffolds were studied preliminarily. Typically, PLCL/PDA scaffolds and PLCL/BG2/PDA scaffolds were pretreated for 12 h with 5 M sodium hydroxide (NaOH). Then the scaffolds were submerged in individual tubes with 5 mL PBS (at pH 7.4). The tubes were maintained at 37℃ in an incubator for 7 and 14 days. The mass loss, compression modulus, the pH value of the degradation medium and the surface morphology of the PDA coating scaffolds were measured.


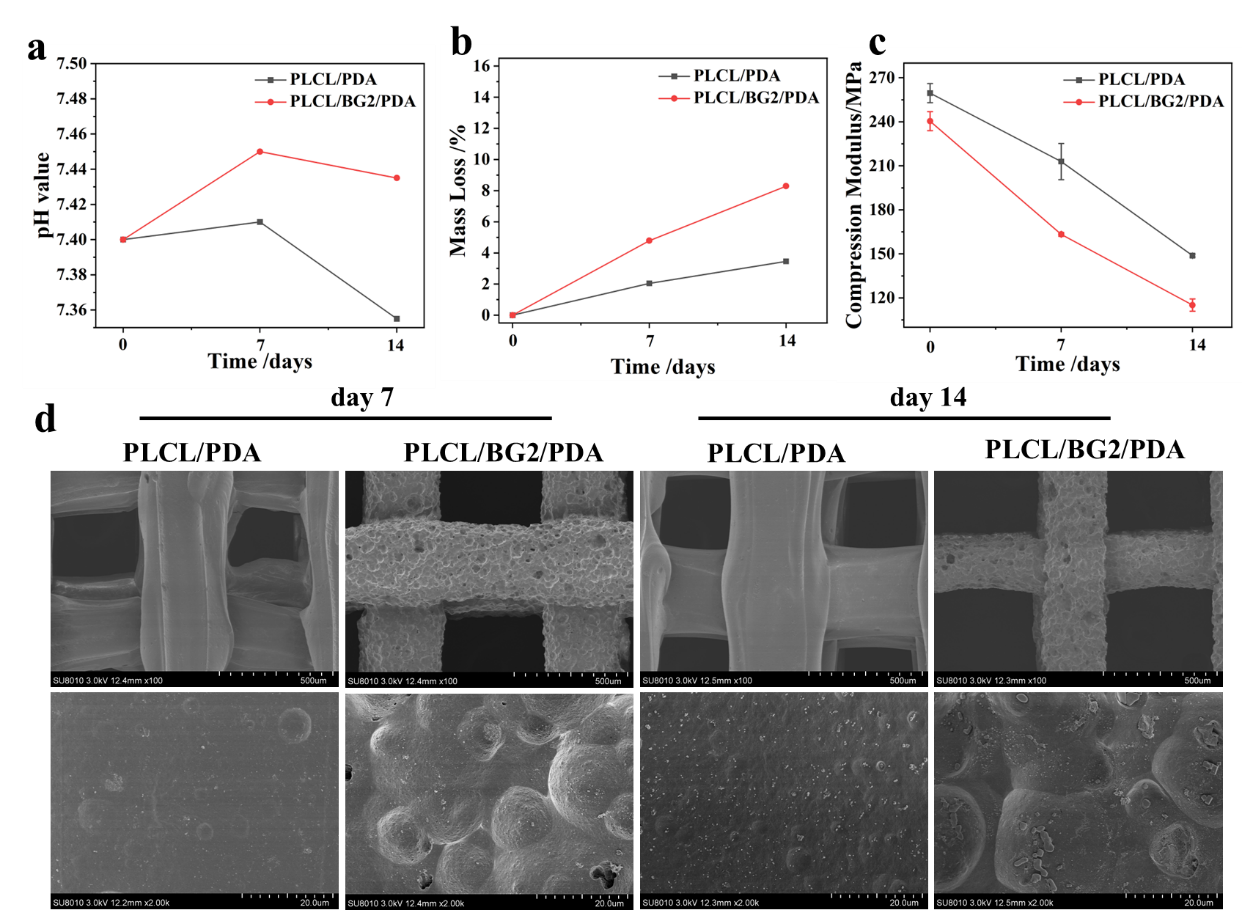


**Fig. S9**. Degradation propertied of sodium hydroxide treatment scaffolds. (a) The pH value of the degradation medium of scaffolds. (b) Mass loss of scaffolds. (c) Compression modulus of scaffolds. (d) Gross morphology of scaffolds.

The pH values of both two groups were slightly rising after 7days immersion, and then showing a downward trend (**Fig. S9**a). The mass loss of the treated groups showed a rapid mass loss rising trend from 0 to 14 days (**Fig. S9**b). The compression modulus of two groups both decreased significantly (**Fig. S9**c). After 7 days and 14 days degradation in PBS at 37℃, the overall gross morphologies of PLCL/PDA scaffolds showed no visual differences compared to the day the experiments (**Fig. S9**d). However, the surface of sodium hydroxide predegraded PLCL/BG2/PDA scaffolds was rough considerably. It attributed to the sodium hydroxide and BG, which rendered the surface of PLCL/BG2/PDA scaffold more hydrophilic for cell attachment. The results discussed above indicated that sodium hydroxide treatment might aggravate degradation of scaffolds. Degradation of polymer is complex, deep and detailed research is needed.
